# Oncogenic Transformation by Inhibitor-Sensitive and -Resistant EGFR Mutants

**DOI:** 10.1371/journal.pmed.0020313

**Published:** 2005-10-04

**Authors:** Heidi Greulich, Tzu-Hsiu Chen, Whei Feng, Pasi A Jänne, James V Alvarez, Mauro Zappaterra, Sara E Bulmer, David A Frank, William C Hahn, William R Sellers, Matthew Meyerson

**Affiliations:** 1Department of Medical Oncology, Dana-Farber Cancer Institute, Boston, Massachusetts, United States of America,; **2**Department of Medicine, Brigham and Women's Hospital and Harvard Medical School, Boston, Massachusetts, United States of America,; **3**The Broad Institute of Harvard and MIT, Cambridge, Massachusetts, United States of America,; **4**Department of Pathology, Harvard Medical School, Boston, Massachusetts, United States of America; Memorial-Sloan Kettering Cancer CenterUnited States of America

## Abstract

**Background:**

Somatic mutations in the kinase domain of the epidermal growth factor receptor tyrosine kinase gene *EGFR* are common in lung adenocarcinoma. The presence of mutations correlates with tumor sensitivity to the EGFR inhibitors erlotinib and gefitinib, but the transforming potential of specific mutations and their relationship to drug sensitivity have not been described.

**Methods and Findings:**

Here, we demonstrate that *EGFR* active site mutants are oncogenic. Mutant *EGFR* can transform both fibroblasts and lung epithelial cells in the absence of exogenous epidermal growth factor, as evidenced by anchorage-independent growth, focus formation, and tumor formation in immunocompromised mice. Transformation is associated with constitutive autophosphorylation of EGFR, Shc phosphorylation, and STAT pathway activation. Whereas transformation by most *EGFR* mutants confers on cells sensitivity to erlotinib and gefitinib, transformation by an exon 20 insertion makes cells resistant to these inhibitors but more sensitive to the irreversible inhibitor CL-387,785.

**Conclusion:**

Oncogenic transformation of cells by different *EGFR* mutants causes differential sensitivity to gefitinib and erlotinib. Treatment of lung cancers harboring *EGFR* exon 20 insertions may therefore require the development of alternative kinase inhibition strategies.

## Abstract 2

Different EGFR mutations are associated with lung cancer. All of the classes can transform fibroblasts and lung epithelial cells, most are sensitive to erlotinib and gefininib, but exon 20 mutations are only sensitive to an irreversible EGFR inhibitor.

## Introduction

The human epidermal growth factor receptor gene product (EGFR), a member of the ErbB family of receptor tyrosine kinases, is an integral component of signaling in epithelial cell proliferation. Stimulation of the receptor with EGF or other cognate ligands induces receptor dimerization and autophosphorylation, providing docking sites for SH2-containing adaptor proteins that mediate the activation of intracellular signaling pathways [1–3].

Consistent with a role in proliferative signaling, the oncogenic potential of *EGFR* variants with deletions in the extracellular domain, including the *v-erbB* oncogene of avian erythroblastosis virus and the vIII mutant found in human cancers, transforms vertebrate cells in the absence of exogenous EGF [4–7]. In contrast, overexpression of the wild-type *EGFR* gene can transform NIH-3T3 cells only upon EGF addition [8]. Kinase activity is required for ligand-independent transformation by both types of EGFR extracellular domain deletion mutant [9,10].

A series of novel *EGFR* kinase domain mutations observed in human lung adenocarcinomas has recently been described [11–16]. These mutations arise in four exons: substitutions for G719 in the nucleotide-binding loop of exon 18, in-frame deletions within exon 19, in-frame insertions within exon 20, and substitutions for L858 or L861 in the activation loop in exon 21. Tumors from patients with clinical responses to the EGFR inhibitors gefitinib or erlotinib have been shown to contain *EGFR* deletion mutations or substitution mutations [11,12,13,15], but no exon 20 insertion mutations have been reported in this group of clinical responders. Although exon 20 mutations were not widely reported at first, recently five large-scale studies that sequenced *EGFR* exons 18 through 21 reported a total of 18 exon 20 insertions out of 350 *EGFR* mutations identified in 1,108 non-small-cell lung cancers [14–18]. Patients who responded to gefitinib and subsequently relapsed were found to have T790M secondary mutations, also in exon 20 [19,20].

Although gefitinib treatment and small interfering RNA experiments suggest that cells expressing mutant *EGFR* are dependent on EGFR function for survival [11,21,22], the direct transforming potential of the mutations observed in lung adenocarcinoma has not been described. Here, we assess the ability of these *EGFR* kinase domain mutations to constitutively activate EGFR signaling and contribute to tumorigenesis in model cell culture systems.

## Methods

### Cell Culture

NIH-3T3 cells obtained fromATCC (Manassas, Virginia, United States) were maintained in DMEM (Cellgro/Mediatech, Herndon, Virginia, United States) supplemented with 10% calf serum (Gibco/Invitrogen, Carlsbad, California, United States) and penicillin/streptomycin (Gibco/Invitrogen). NCI-H3255 cells were maintained in ACL-4 media as previously described [11]. Unless otherwise noted, cells were placed in media containing 0.5% calf serum 24 h prior to EGF (Biosource, Camarillo, California, United States) stimulation. hTBE cells expressing SV40 small T and large T antigens and the human telomerase catalytic subunit hTERT were maintained in serum-free, defined medium as described [23]. Neutralizing antibodies were added 3 h prior to EGF stimulation at the following concentrations: 12 μg/ml anti-EGF (R&D Systems, Minneapolis, Minnesota, United States; #MAB636), 12 μg/ml anti-TGFα (R&D Systems; #AF-239-NA), and 12 μg/ml anti-EGFR (Upstate, Waltham, Massachusetts, United States; #05–101). Gefitinib and erlotinib were purchased from WuXi Pharmatech (Shanghai, China) and diluted in DMSO to the indicated concentrations. CL-387,785 was purchased from Calbiochem (San Diego, California, United States) and diluted in DMSO to the indicated concentrations.

### Expression Constructs

*EGFR* was amplified from a cDNA template with the PCR primers 5′-GATGATATCATGCGACCCTCCGGGAC-3′ and 5′-ATCGATATCTCATGCTCCAATAAATTC-3′, digested with EcoRV, and inserted into the SnaB1 site of pBabe-Puro. Point mutations, insertions, and deletions were made using the Quick-Change Mutagenesis XL kit (Stratagene, La Jolla, California, United States) with the following oligonucleotide primers: 5′-AAGATCACAGATTTTGGGAGGGCCAAACTGCTGGGTG-3′ and 5′-CACCCAGCAGTTTGGCCCTCCCAAAATCTGTGATCTT-3′ for L858R; 5′-AAGATCAAAGTGCTGAGCTCCGGTGCGTTCG-3′ and 5′-CGAACGCACCGGAGCTCAGCACTTTGATCTT-3′ for G719S; 5′-GGTGCACCGCGCCCTGGCAGCCA-3′ and 5′-TGGCTGCCAGGGCGCGGTGCACC-3′ for D837A; 5′-GTCGCTATCAAGGAACCAACATCTCCGAAA-3′ and 5′-TTTCGGAGATGTTGGTTCCTTGATAGCGAC-3′ for L747_E749del, A750P; 5′-GGCCAGCGTGGACAACCCCGGCAACCCCCACGT-3′ and 5′-ACGTGGGGGTTGCCGGGGTTGTCCACGCTGGCC-3′ for D770_N771insNPG. All constructs were fully sequenced.

### Transfection and Infection

Replication incompetent retroviruses were produced from pBabe-Puro-based vectors either by cotransfection of 293T cells with pCL-Ampho (Imgenex, San Diego, California, United States) or by transfection into the Phoenix 293T packaging cell line (Orbigen, San Diego, California, United States) using Lipofectamine 2000 (Invitrogen). Cells were infected with these retroviruses in the presence of polybrene. Two days after infection, puromycin (2 μg/ml for NIH-3T3s or 0.5 μg/ml for hTBE cells; Sigma, St. Louis, Missouri, United States) was added and pooled stable cell lines were selected, from which clonal cell lines were derived.

### Soft Agar Anchorage-Independent Growth Assay

EGFR-expressing NIH-3T3 cells were suspended in a top layer of DMEM containing 10% calf serum and 0.4% Select agar (Gibco/Invitrogen) and plated on a bottom layer of DMEM containing 10% calf serum and 0.5% Select agar. EGF, gefitinib, or erlotinib was added as described to the top agar. NIH-3T3 colonies were counted in triplicate wells from ten fields photographed with a 10× objective. Growth of hTBE cells in soft agar was determined by plating 1 × 10^5^ cells in triplicate in 0.4% Noble agar. Colonies of hTBE cells were counted microscopically 6–8 wk after plating with the MultiImage imaging counter (Alpha Innotech, San Leandro, California, United States).

### Focus Formation Assay

NIH-3T3 cells infected with EGFR retrovirus diluted 1:1, 1:10, or 1:100 were split 2 d after infection into 10-cm plates with and without puromycin selection. Cells for the focus assay were maintained without passage as a monolayer in the absence of puromycin for 3 wk, after which foci were stained with crystal violet (Accustain; Sigma) and scored. Numbers of foci were multiplied by the viral dilution factor and normalized to the relative number of infectious units in each viral stock, as determined by a WST assay (Roche, Basel, Switzerland) on cells after 3 d of puromycin selection.

### Nude Mouse Injection

In this assay, 2 × 10^6^ cells were injected subcutaneously into immunocompromised mice, three injections per animal, as described [24]. Tumors were counted and tumor diameter was measured after 5 wk. Standard error of the mean is indicated.

### Immunoblotting

Cells were lysed in a buffer containing 50 mM Tris-HCl (pH 7.4), 150 mM NaCl, 2.5 mM EDTA, 1% Triton X-100, and 0.25% IPEGAL. Protease inhibitors (Roche) and phosphatase inhibitors (Calbiochem) were added prior to use. Samples were normalized for total protein content unless otherwise indicated. Lysates were boiled in sample buffer, separated by SDS-PAGE on 8% or 10% polyacrylamide gels, transferred to nitrocellulose, and probed as described. Antibodies used for immunoblotting were: anti-EGFR (#2232, Cell Signaling Technologies), anti-phospho-EGFR Y1173 (#05–483, Upstate), anti-phospho-EGFR Y1068 (#2234, Cell Signaling Technologies), anti-phospho-EGFR Y845 (Cell Signaling Technologies, Beverly, Massachusetts, United States; #2231), anti-phospho-EGFR Y1045 (Cell Signaling Technologies; #2237), anti-actin (Santa Cruz Biotechnology, Santa Cruz, California, United States; #sc-1615), anti-Shc (Upstate; #06–203), anti-phospho-Shc Y317 (Upstate; #05–668), anti-Stat3 (Cell Signaling Technologies; #9132), and anti-phospho-Stat3 Y705 (Cell Signaling Technologies; #9131), anti-phospho-Akt S473 (Cell Signaling Technologies; #9271), and anti-Akt (Cell Signaling Technologies; #9272).

### Immunoprecipitation

Cells were lysed as described above. Anti-EGFR conjugated beads (Santa Cruz Biotechnology; #sc-120AC) were used for immunoprecipitation. Beads (25 μl) were incubated with fresh lysate in 300 μl of lysis buffer for 1 h, washed twice with lysis buffer, eluted in 1% SDS at room temperature for 20 min, and boiled in sample buffer.

### Luciferase Assay

The STAT3 (signal transducer and activator of transcription 3) reporter m67 pTATA TK-luc [25] was kindly provided by J. Bromberg, and the Renilla luciferase reporter phRL tk-luc was purchased from Promega (Madison, Wisconsin, United States). NIH-3T3 cells, infected with EGFR or control retroviruses, were plated on 24-well plates and transfected with 2 μg of m67-luc and 0.2 μg phRL tk-luc with Lipofectamine 2000 (Invitrogen). After 48 h, cells were lysed and luminescence was measured using the dual-luciferase reagents from Promega, according to the manufacturer's instructions, using a Luminoskan Ascent luminometer (ThermoLab Systems, Helsinki, Finland). STAT3-dependent luciferase production was normalized to chemiluminescence values from the control Renilla luciferase.

## Results

### Expression of Mutant *EGFR* Induces Oncogenic Transformation

To assess the oncogenic potential of *EGFR* kinase domain mutants, tumor-derived mutations were introduced into the wild-type human *EGFR* cDNA by site-directed mutagenesis. The resulting wild-type and mutant *EGFR* cDNAs were then cloned into the pBabe-Puro retroviral vector and transferred into NIH-3T3 cells by retroviral infection. We initially examined two representative substitution mutations: G719S, observed in exon 18, and L858R, observed in exon 21 (Figure 1). The L858R and G719S mutants were able to transform NIH-3T3 cells to anchorage independence in the absence of exogenous EGF, as assayed by colony formation in soft agar (Figure 1A, top photographs). In contrast, as previously described [8], wild-type *EGFR* transformed only upon EGF addition (Figure 1A, bottom photographs). The kinase-dead D837A mutant [9], included as a negative control, failed to induce colony formation in the presence or absence of EGF. EGFR expression levels were approximately equal for each pooled stably-transfected cell population (Figure 1B). Clonal cell lines derived from the pooled stably-transfected cells expressing the mutant EGFR exhibited profound morphological alterations characterized by a fusiform, refractile phenotype (unpublished data). Levels of L858R EGFR expression necessary to achieve transformation in this model cell culture system were no higher than expression levels observed in the human lung adenocarcinoma cell line H3255 bearing the L858R mutation (Figure 1C).

**Figure 1:**
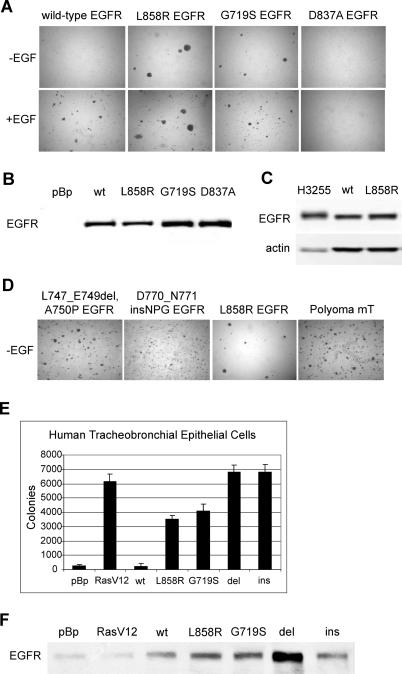
Mammalian Cells Expressing the Lung Cancer-Derived Mutant EGFR Grow in an Anchorage-Independent Manner (A) NIH-3T3 cells infected with retroviruses encoding the indicated wild-type or mutant *EGFR* were selected in the presence of 2.5 μg /ml puromycin for 4 d. In the top photomicrographs, 1 × 10^5^ cells were suspended in soft agar for a colony formation assay and photographed after 3 wk incubation at 37 °C. Expression of lung cancer-derived missense *EGFR* mutants, but not wild-type or kinase-inactive D837A *EGFR,* induces colony formation in soft agar. In the bottom photomicrographs, samples were identical, but 20 ng/ml EGF was added to the top agar. Representative photomicrographs are shown. (B) Anti-EGFR immunoblot analysis of pooled stable NIH-3T3 cells infected as described in (A). All *EGFR* constructs are expressed at similar levels. pBp, pBabe-Puro vector; wt, wild-type EGFR. (C) Lysates from 4 × 10^4^ cells from the human lung adenocarcinoma cell line H3255, harboring the L858R mutation in *EGFR,* or the wild-type or L858R *EGFR*-overexpressing NIH-3T3 cells, were immunoblotted for total *EGFR* levels. Although total protein levels per cell are lower for the H3255 than the NIH-3T3 cells, EGFR expression levels are slightly higher in the H3255s. (D) NIH-3T3 cells infected with retroviruses encoding the mutant *EGFR* were selected in the presence of 2 μg/ml puromycin for 9 d. Selected cells (1 × 10^5^) were suspended in soft agar for a colony formation assay and photographed after 3 wk incubation at 37 °C. Expression of the deletion and insertion *EGFR* mutants induced formation of colonies in soft agar with higher efficiency than expression of L858R. Representative photos are shown. Polyoma mT, NIH-3T3 cells infected with positive control pBabe-Puro retrovirus encoding the polyoma middle T antigen. (E) hTBE cells expressing the SV40 early region and hTERT were infected with control virus pBabe-Puro (pBp) or with viruses encoding the indicated *EGFR* alleles. Cells were plated in 0.4% Noble agar, and colonies were counted with an automated imager at 6 wk. Mean ± standard deviation is shown for three independent determinations. Control cells (pBp) formed many microscopic colonies, but colonies formed by cells expressing *EGFR* mutants were more numerous and larger. del, L747_E749del A750P mutated EGFR; ins, D770_N771insNPG mutated EGFR; pBp, pBabe-Puro vector; RasV12, V12 H-Ras; wt, wild-type EGFR. (F) Anti-EGFR immunoblot analysis of hTBE cells infected as described in (E). All *EGFR* constructs were expressed at similar levels. pBp, pBabe-Puro vector; wt, wild-type EGFR.

Transformation of NIH-3T3 cells by L858R or G719S EGFR was further assessed in two independent assays. Expression of the EGFR point mutants in NIH-3T3 cells caused loss of contact inhibition, resulting in focus formation on an unselected monolayer, whereas the wild-type and D837A kinase-inactive controls did not (Table 1). In addition, injection of clonal, transformed NIH-3T3 fibroblasts expressing L858R and G719S *EGFR* into immunocompromised mice led to the formation of tumors (Table 2). No tumor formation was observed upon injection of cells infected with vector, wild-type, and kinase-dead controls.

**Table 1:**
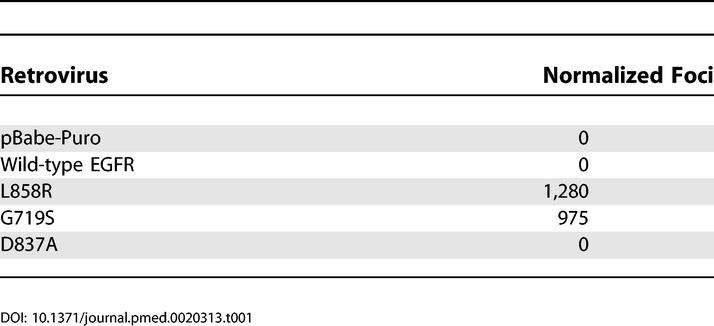
NIH-3T3 Cells Expressing the Lung Cancer-Derived Mutant EGFR Display Loss of Contact Inhibition

**Table 2:**
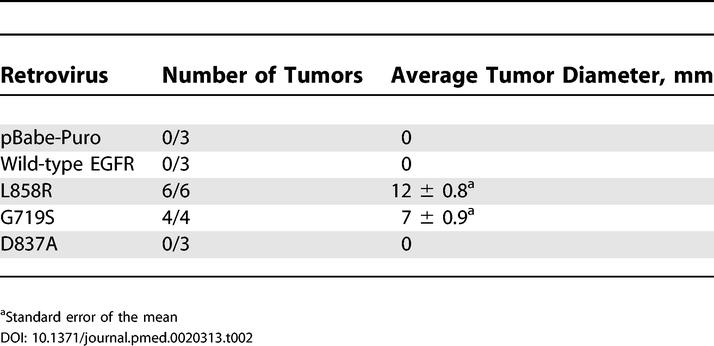
Clonal NIH-3T3 Cell Lines Expressing the Lung Cancer-Derived Mutant EGFR Form Tumors in Immunocompromised Mice

Representative exon 19 deletion and exon 20 insertion mutations of *EGFR* were then assessed for transforming activity. Mutants L747_E749del, A750P [11] and D770_N771insNPG (K. Naoki and M. M., unpublished data) were introduced into NIH-3T3 cells by retrovirus-mediated gene transfer as described above. Cells expressing the *EGFR* deletion and insertion mutants formed colonies in soft agar with a higher efficiency than that of cells expressing the missense mutants, comparable to the colony formation efficiency of cells expressing polyoma middle T antigen (Figure 1D). Expression of the deletion mutant was comparable to that of L858R EGFR, whereas expression of the insertion mutant was lower, as reflected in the *EGFR* expression levels of the clonal cell lines (Figure 2A and unpublished data). Cells expressing the L747_E749del A750P and D770_N771insNPG *EGFR* mutants also exhibited a greater degree of loss of contact inhibition than was observed in cells expressing the L858R or G719S EGFR mutants in a primary focus formation assay (unpublished data).

**Figure 2:**
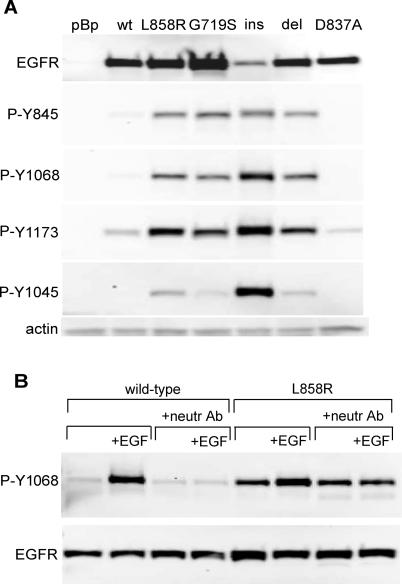
Ligand-Independent Activation of the Mutant EGFR (A) Cells expressing the wild-type or mutant *EGFR* were lysed and immunoblotted with antibodies to total EGFR or antibodies that recognize specific phosphorylation sites in the EGFR C-terminal tail as labeled. All four mutant EGFR proteins, representative of the four classes of *EGFR* mutations observed in lung adenocarcinoma tumor DNA, exhibited constitutive phosphorylation on the indicated C-terminal autophosphorylation sites. Note that the nomenclature for the anti-phospho-EGFR antibodies reflects elimination of the 24-amino acid signal peptide. Due to difficulties in isolating clonal cell lines with the same levels of mutant EGFR expression, G719S is expressed at higher levels and D770_N771ins NPG at lower levels than the other mutant EGFR. del, L747_E749del A750P; ins, D770_N771insNPG; pBp, pBabe-Puro vector control; wt, wild-type EGFR. (B) Cells expressing the wild-type or L858R *EGFR* were placed in media containing 0.5% CS for 24 h. A combination of three neutralizing antibodies (anti-EGF, anti-TGFα, and anti-EGFR) was added 3 h prior to EGF stimulation and lysis. Upper row of blots show the anti-phospho-EGFR Y1068 immunoblots. The lower row shows anti-EGFR immunoblots. No inhibition of L858R EGFR autophosphorylation was observed upon treatment with a combination of three neutralizing antibodies (“neutr Ab”) sufficient to prevent EGF stimulation of autophosphorylation of the wild-type EGFR.

To determine the ability of mutant *EGFR* to transform a more physiologically relevant cell type, retroviruses expressing the L858R and G719S mutant forms of *EGFR* were introduced into hTBE cells expressing the SV40 early region T antigens and the human telomerase catalytic subunit hTERT [23]. We previously showed that such cells are fully transformed by the additional expression of oncogenic alleles of H- or K-RAS [23]. Similarly, the expression of L858R and G719S mutant *EGFR* genes conferred enhanced anchorage-independent growth upon such hTBE cells, with colony numbers approximately 15-fold above the background level of microscopic colonies observed in hTBE cells expressing wild-type *EGFR* or a control vector (Figure 1E). The representative deletion and insertion mutants, L747_E749del A750P and D770_N771insNPG, formed colonies in soft agar with even greater efficiency, with the caveat that the deletion mutant is expressed at higher levels than the other mutants in this assay (Figure 1F). Similar to hTBE cells expressing H-RAS V12, expression of these EGFR mutants did not increase the rate of cell proliferation in defined medium (unpublished data).

Multiple tumor-derived mutants of *EGFR* therefore contribute to oncogenic transformation as shown by three different assays: anchorage-independent cell growth, focus formation, and in vivo tumor formation.

### Mutant EGFR Proteins Are Constitutively Active

To determine whether transformation by mutant *EGFR* is associated with constitutive receptor activation in the absence of exogenous EGF, tyrosine autophosphorylation in the C-terminal tail of EGFR was examined by immunoblotting of cell lysates. Constitutive tyrosine phosphorylation of the mutant EGFR molecules was observed at several C-terminal sites, including Y845, Y1068, and Y1173 (Figure 2A). High-level phosphorylation of the insertion mutant at Y1045, the docking site for the Cbl E3 ubiquitin ligase [26], is correlated with decreased abundance of this protein (Figure 2A), but whether the differential protein levels are a result of Cbl activity has not been confirmed.

The constitutive increase in EGFR activity appears to be ligand-independent, as a combination of neutralizing antibodies against EGF, TGFα, and EGFR failed to inhibit elevated basal levels of L858R autophosphorylation (Figure 2B). However, tyrosine phosphorylation on the EGFR mutants could be further increased by EGF stimulation (Figure 2B), suggesting that the mutant EGFRs exhibit both ligand-independent and ligand-dependent activation, similar to that observed upon EGF stimulation of the L858R mutant H3255 lung adenocarcinoma cell line [21]. Ligand-independent activation of EGFR with lung cancer-derived kinase domain mutations has not been observed by other groups working with transient transfection systems [22,27]. We too have failed to detect constitutive elevation of mutant receptor autophosphorylation when transiently expressed in NIH-3T3 and HeLa cells (unpublished data). The reason for this phenotypic difference remains unclear.

### Expression of Mutant EGFR Results in Activation of Shc, STAT3, and Akt

We next asked whether constitutive activation of mutant EGFR is associated with alterations in downstream signaling pathways. Because Y1173, a docking site for the adaptor protein Shc [28], is constitutively phosphorylated on mutant EGFR (Figure 2A), we analyzed Shc-EGFR complex formation in cells expressing wild-type and mutant EGFR. Coimmunoprecipitation studies revealed a low level of constitutive Shc binding to the L858R EGFR, further augmented by EGF stimulation (Figure 3A), whereas Shc complexed with the wild-type EGFR only upon EGF stimulation. Immunoblotting of whole cell lysates with an antibody specific for tyrosine-phosphorylated Shc revealed constitutive phosphorylation on Shc in cells expressing the L858R EGFR, consistent with the known phosphorylation of EGFR-bound Shc [29]; in contrast, in cells expressing wild-type EGFR, Shc was phosphorylated only in response to EGF stimulation (Figure 3B). Similar to the situation with receptor autophosphorylation, constitutive phosphorylation of Shc in mutant EGFR-expressing cells has not been observed in transient expression systems [27].

**Figure 3:**
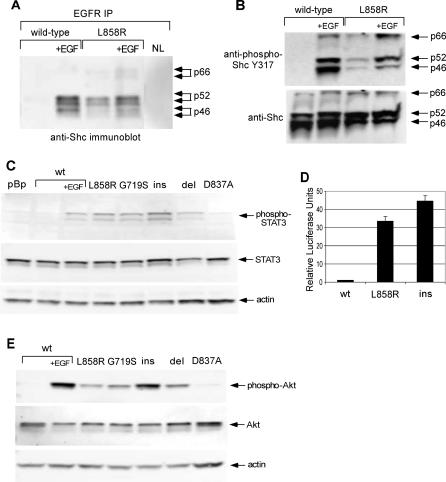
Shc and STAT3 Signaling Pathways Are Constitutively Activated in Cells Expressing the Mutant EGFR (A) Cells expressing the wild-type or L858R mutant *EGFR* were placed in 0.5% calf serum for 24 h and left unstimulated or stimulated with 20 ng/ml EGF (“+EGF”) for 8 min. EGFR was immunoprecipitated from 200 μg of cell lysate, and eluted immune complexes were separated by SDS-PAGE and immunoblotted with anti-Shc. Shc constitutively coimmunoprecipitates with the L858R EGFR but not the wild-type EGFR. IP, immunoprecipitation; NL, no-lysate control immunoprecipitation. (B) Anti-phospho-Shc immunoblots (upper row of blots) and anti-Shc immunoblots (lower row) of whole cell lysates from the experiment in (A). All three Shc isoforms are constitutively phosphorylated in cells expressing the L858R *EGFR*. (C) Immunoblots of whole cell lysates with anti-phospho-STAT3 Y705 (upper row), total STAT3 (middle row), or actin as a loading control (lower row). STAT3 is constitutively phosphorylated in cells expressing any of the four mutant EGFR proteins, representative of the four classes of *EGFR* mutations observed in lung adenocarcinoma tumor DNA. del, L747_E749del A750P; ins, D770_N771insNPG; pBp, pBabe-Puro vector control; wt, wild-type EGFR. (D) M67 STAT3 luciferase reporter assay. NIH-3T3 cells expressing the indicated EGFR were transfected with a STAT-dependent reporter (m67-firefly luciferase) [25] and a control reporter expressing Renilla luciferase. STAT-dependent luciferase production was measured after 48 h and normalized to Renilla luciferase. The normalized luciferase values were divided by the values for cells expressing the wild-type EGFR to produce relative luciferase units. NIH-3T3 expressing mutant forms of EGFR exhibited elevated levels of STAT-dependent transcriptional activity relative to wild-type. ins, D770_N771insNPG EGFR; wt, wild-type EGFR. (E) Immunoblots of whole cell lysates with anti-phospho-Akt S473 (upper row), total Akt (middle row), or actin as a loading control (lower row). Akt is constitutively phosphorylated in cells expressing mutant EGFR. del, L747_E749del A750P; ins, D770_N771insNPG; wt, wild-type EGFR.

Consistent with previous reports on L858R mutant EGFR [22], STAT signaling pathways are constitutively activated in the transformed NIH-3T3 cells. Immunoblotting with antibodies specific for phosphorylated Y705, the tyrosine responsible for STAT3 dimerization [30], revealed constitutive phosphorylation in cells expressing the lung cancer-derived mutant EGFR but not wild-type EGFR (Figure 3C). Increased STAT3-dependent gene expression in cells expressing the mutant EGFRs was confirmed in a reporter assay (Figure 3D) using a STAT-dependent luciferase construct [25].

Constitutive phosphorylation of mutant EGFR on Y1068 (see Figure 2A), the binding site for the phosphatidylinositol 3-kinase interacting protein Gab1 [31], indicated that signaling pathways downstream of phosphatidylinositol 3-kinase might be constitutively activated as well. One such pathway is controlled by the serine/threonine kinase Akt, which is involved in promotion of cell survival. Western blotting with anti-phospho-Akt confirmed that Akt is constitutively activated in cells expressing the mutant EGFR (Figure 3E). We therefore conclude that at least a subset of physiological EGFR signaling pathways is activated by stable expression of mutant *EGFR*.

### Transformation by the Exon 20 Insertion Mutant Is Not Sensitive to Gefitinib or Erlotinib

Given the association between the presence of activating *EGFR* mutations and clinical responses to gefitinib or erlotinib in lung adenocarcinoma patients [11,12,13,15], we assessed the ability of these EGFR inhibitors to inhibit anchorage-independent growth of clonal NIH-3T3 cell lines expressing wild-type or mutant *EGFR*. Consistent with the increased sensitivity to gefitinib and erlotinib of patient tumors harboring the missense mutations or exon 19 deletions, anchorage-independent growth of cells expressing L858R, G719S, or L747_E749del A750P was inhibited by 100 nM erlotinib (Figure 4A and 4B) or gefitinib (Figure 4B and unpublished data), although the G719S mutant may be somewhat more resistant to gefitinib (Figure 4A and unpublished data), consistent with other in vitro studies [32]. In contrast, 1 μM erlotinib (Figure 4A) or gefitinib (unpublished data) did not inhibit anchorage-independent growth of EGF-treated cells overexpressing the wild-type *EGFR*.

**Figure 4:**
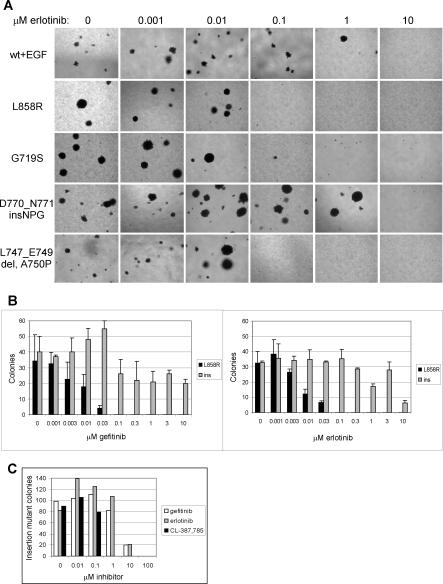
Sensitivity of Cell Transformation Induced by Expression of Mutant *EGFR* Characterized by Missense Mutation or Exon 19 Deletion, but not Exon 20 Insertion, to Gefitinib and Erlotinib (A) Anchorage-independent growth of clonal NIH-3T3 cells transformed with mutant *EGFR* or EGF-stimulated wild-type *EGFR* treated with the indicated concentrations of erlotinib immediately prior to suspension in soft agar. Transformation induced by expression of L858R, G719S, and L747_E749del A750P *EGFR,* but not EGF-stimulated wild-type *EGFR* or D770_N771insNPG *EGFR,* was inhibited by 0.1 μM erlotinib. Representative photographs are shown. (B) Number of colonies formed in soft agar by clonal NIH-3T3 cells expressing L858R *EGFR* and D770_N771insNPG *EGFR* treated with the indicated concentrations of gefitinib or erlotinib immediately prior to suspension in soft agar. Transformation by cells expressing the L858R *EGFR* was inhibited by 0.1 μM gefitinib or erlotinib, whereas transformation by cells expressing the insertion mutant was resistant to low concentrations of these inhibitors. Colonies were quantitated by counting ten fields each of triplicate wells photographed with a 10× objective; mean ± standard deviation is shown. Ins, D770_N771insNPG *EGFR*. (C) Transformation induced by expression of D770_N771insNPG *EGFR* is inhibited 10-fold more efficiently by the irreversible EGFR inhibitor CL-387,785 [35]. Clonal NIH-3T3 cells expressing the insertion mutant were treated with the indicated concentrations of gefitinib, erlotinib, or CL-387,785 immediately prior to suspension in soft agar. This assay was not done in triplicate, but the results are representative of two independent experiments. The number of colonies was normalized to maximum colony formation for each treatment, and sigmoidal dose response curves were fitted to the data using Prism Graphpad software to determine IC50s.

Transformation by the D770_N771insNPG *EGFR* mutant was remarkably insensitive to gefitinib and erlotinib, as inhibition of colony growth in soft agar required exposure to 100-fold higher concentrations (>1 μM) of these agents than was required to inhibit colony formation by cells expressing the *EGFR* missense mutants or deletion mutant (Figure 4A). In fact, no significant inhibition of anchorage-independent growth of cells expressing D770_N771insNPG *EGFR* was observed at 3 μM gefitinib or erlotinib (Figure 4B). The concentrations of gefitinib or erlotinib necessary to reverse insertion mutant transformation are therefore higher than the achievable serum concentrations of gefitinib (0.5–1.0 μM) and possibly higher than the achievable serum concentrations of erlotinib (2.8–4.0 μM) [33,34].

Consistent with this result, all three lung adenocarcinoma patients with known exon 20 insertion mutants of *EGFR* have failed to show a clinical response to treatment and have instead achieved only stable disease with erlotinib alone (*n* = 1; L. Sequist and T. Lynch, personal communication), or in combination with chemotherapy (*n* = 2; D. Eberhard and K. Hillan, personal communication). These results suggest that cancers harboring distinct activating kinase domain mutations of *EGFR* may exhibit a differential sensitivity to specific EGFR inhibitors.

Interestingly, the irreversible EGFR inhibitor CL-387,785 [35] is more effective than gefitinib or erlotinib for inhibition of colony formation by cells expressing the exon 20 insertion mutant (Figure 4C). Calculated IC50 values for gefitinib, erlotinib, and CL-387,785 against D770_N771insNPG were 2.6 μM, 2.5 μM, and 0.2 μM, respectively. CL-387,785 had an even greater effect on colony formation by cells expressing L858R *EGFR,* completely inhibiting transformation at 0.003 μM (unpublished data). These effects are also observed upon assessment of receptor autophosphorylation (Figure 5). Although the inhibitory concentrations do not exactly correlate with the results of the colony formation assay, probably due to the difference in duration of the assays, the trends are the same. Insertion mutant autophosphorylation is less sensitive to inhibition by gefitinib than that of L858R, but CL-387,785 is more effective than gefitinib at inhibiting insertion mutant (and L858R) autophosphorylation.

**Figure 5:**
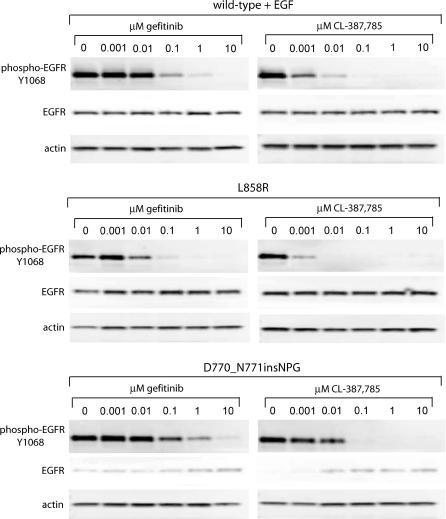
Sensitivity of Mutant EGFR Autophosphorylation to EGFR Inhibitors Reflects Inhibition of Anchorage-Independent Growth Cells expressing wild-type, L858R, or D770_N771insNPG *EGFR* were treated for 2 h with the indicated concentrations of gefitinib or CL-387,785. Cells expressing the wild-type EGFR were then stimulated for 10 min with 7 ng/ml EGF, and all plates were lysed. Whole-cell lysates were immunoblotted for phospho-EGFR Y1068 (upper row of blots), total EGFR (middle row), and actin as a loading control (lower row). Although compound concentrations necessary for inhibition of autophosphorylation do not exactly correspond to inhibition of anchorage-independent growth, the relative sensitivity of autophosphorylation of the wild-type and mutant EGFR to gefitinib or CL-387,785 mirrors the relative sensitivity of colony formation to these inhibitors.

## Discussion

Treatment with the EGFR inhibitors gefitinib and erlotinib has led to dramatic responses in many lung cancer patients, predominantly for those cancers in which *EGFR* mutations can be detected. However, there has been a subset of lung cancer patients with these mutations who do not respond to the EGFR inhibitors in current clinical use.

By demonstrating that lung-cancer derived kinase domain mutants of *EGFR* are constitutively activated and that they can transform cultured mammalian cells, we have provided an in vitro system with which to study EGFR-dependent oncogenesis in a genetically homogeneous background. Although the anchorage-independent growth assay measures only one of many phenotypes of transformation and does not, for example, recapitulate tumor microenvironment or account for the influence of the immune system on tumor formation, this system will be useful for dissecting inhibitor response and downstream signaling pathways, particularly for those mutants not found in existing cancer-derived cell lines.

Using the NIH-3T3 transformation system, we have found that transformation by an exon 20 insertion mutant is resistant to inhibition by gefitinib and erlotinib. Strikingly, transformation by this *EGFR* exon 20 insertion mutant is more sensitive to treatment with an irreversible inhibitor, CL-387,785. This compound was previously found to be active against EGFR containing the exon 20 point mutation T790M, associated with resistance to gefitinib and erlotinib [19].

These results indicate a need for the use of novel EGFR inhibitors in primary treatment of lung cancers harboring the exon 20 insertion mutations. Furthermore, the distinct inhibitor sensitivity of various *EGFR* mutants argues that therapies may need to be targeted against specific mutant forms of a protein, whereas generalized inhibition of a particular oncogenic target may not be sufficient.

**Patient Summary**

**Background**

While lung cancer is still one of the deadliest cancers, a new class of drugs called epidermal growth factor receptor (EGFR) inhibitors have shown promising results in some patients. As the name suggests, these drugs inhibit a protein called EGFR, which is altered in a subset of lung cancers.

**What Did the Researchers Do and Find?**

The aim of this study was to understand in more detail what role the different EGFR alterations played in the tumor and which ones made the tumor responsive to EGFR inhibitor treatment. First, the researchers introduced different altered EGFR versions into human cells and studied their behavior. The EGFR protein, which can stimulate cell growth, is normally tightly regulated and active only when a cell receives a signal from its neighbors. However, the alterations made the EGFR protein always active, regardless of a stimulating signal, and caused them to “grow out of control.” They then treated the cells expressing the various alterations with different EGFR inhibitor drugs and showed that the specific alteration determined whether cell growth could be stopped by a specific drug.

**What Do the Results Mean for Patients?**

EGFR inhibitors are still considered to be experimental treatment. Researchers are making progress in understanding how genetic alterations in EGFR cause abnormal growth in some lung cancers and also which specific alterations cause the tumor to be responsive to a particular drug. The goal is to match the tumor and the drug to maximize anti-tumor response and avoid giving a drug that doesn't work with a particular tumor.

**Where Can I Get More Information?**

The following Web sites contain information on lung cancer, the role of EGFR mutations, and the EGFR class of drugs.

Lung Cancer Online (follow links for experimental treatments and EGFR inhibitors):

http://www.lungcanceronline.org/index.htm

US National Cancer Institute information page on lung cancer:

http://www.nci.nih.gov/cancertopics/types/lung

Ongoing clinical trials of EGFR inhibitors:

http://www.clinicaltrials.gov

## Abbreviations

EGFR: epidermal growth factor receptor
hTBE: human tracheobronchial epithelial
STAT: signal transducer and activator of transcription
