# Correction: Oncogenic Transformation by Inhibitor-Sensitive and -Resistant EGFR Mutants

**DOI:** 10.1371/journal.pmed.1004470

**Published:** 2024-09-16

**Authors:** Heidi Greulich, Tzu-Hsiu Chen, Whei Feng, Pasi A. Jänne, James V. Alvarez, Mauro Zappaterra, Sara E. Bulmer, David A. Frank, William C. Hahn, William R. Sellers, Matthew Meyerson

After this article [1] was published, concerns were raised about Fig 4A. Specifically:

- The L858R 1 and 10 μM panels are duplicated.
- The wt+EGF 0, 1 and 10, the L858R 0, 0.001, 0.1, 1 and 10, the G719S 0, 0.001 and 0.1, the D770_N771 insNPG 0 and 0.1, and the L747_E749 del, A750P 0.1, 1 and 10 panels appear to have similar background features.

In response, the first and corresponding author, HG, stated that the L858R 1 μM panel in Fig 4A is incorrect and provided an updated version of Fig 4 here where the L858R 1 μM panel has been replaced with the correct panel from the original experiments. They also stated that any similarities in background would be aberrations in the microscope lens or lighting that apply to all images and provided underlying data from the original experiments (S1–S7 Files) and from repeat experiments (S8–S16 Files).

The first and corresponding author, HG, stated that the raw images for the whole plates used for the assay in Fig 4A are no longer available, but they are available for a repeat experiment (see 10.5061/dryad.nvx0k6f1f). They also stated that for the soft agar assays, kinase-inactive EGFR expressing cells (or parental 3T3 cells) are a reasonable negative control as they form no colonies which can be seen in Fig 1A in [1], and Fig 4A does include cells treated with 10 uM erlotinib which is enough to inhibit all soft agar colony formation.

PLOS received contradictory input on whether the similarities in the backgrounds for some of the panels in Fig 4A would be expected for these microscopy images. An independent expert advised that aberrations in the microscope lens or lighting would appear in all panels, not some. They also reviewed the underlying and repeat data provided and advised there are no concerns and that the data provided support the results reported in [1]. While this issue has not been fully resolved, the *PLOS Medicine* Editors are satisfied that the data provided (S3, S13, S14 Files and 10.5061/dryad.nvx0k6f1f) appear to support the published results.

Questions were also raised about EGFR blot data reported for wild-type + EGF experiments in Fig 5, but PLOS reviewed the data provided for these experiments (S4–S7 Files) and concluded that the data support the published results.

The remainder of the data underlying this article are available from the first and corresponding author, HG.

**Fig 4:**
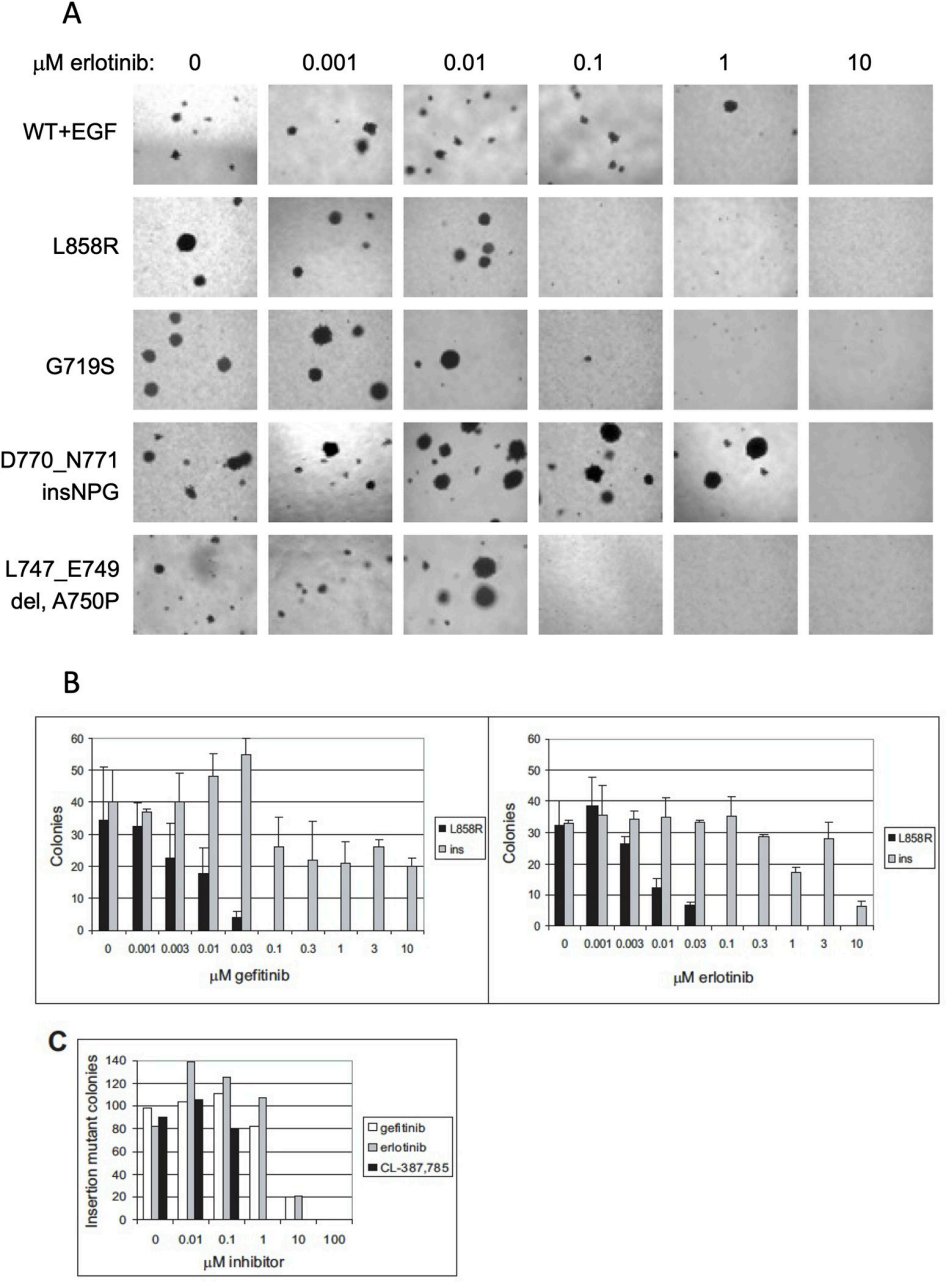
Sensitivity of Cell Transformation Induced by Expression of Mutant EGFR Characterized by Missense Mutation or Exon 19 Deletion, but not Exon 20 Insertion, to Gefitinib and Erlotinib. (A) Anchorage-independent growth of clonal NIH-3T3 cells transformed with mutant EGFR or EGF-stimulated wild-type EGFR treated with the indicated concentrations of erlotinib immediately prior to suspension in soft agar. Transformation induced by expression of L858R, G719S, and L747_E749del A750P EGFR, but not EGF-stimulated wild-type EGFR or D770_N771insNPG EGFR, was inhibited by 0.1 μM erlotinib. Representative photographs are shown. (B) Number of colonies formed in soft agar by clonal NIH-3T3 cells expressing L858R EGFR and D770_N771insNPG EGFR treated with the indicated concentrations of gefitinib or erlotinib immediately prior to suspension in soft agar. Transformation by cells expressing the L858R EGFR was inhibited by 0.1 μM gefitinib or erlotinib, whereas transformation by cells expressing the insertion mutant was resistant to low concentrations of these inhibitors. Colonies were quantitated by counting ten fields each of triplicate wells photographed with a 10x objective; mean ± standard deviation is shown. Ins, D770_N771insNPG EGFR. (C) Transformation induced by expression of D770_N771insNPG EGFR is inhibited 10-fold more efficiently by the irreversible EGFR inhibitor CL-387,785 [35]. Clonal NIH-3T3 cells expressing the insertion mutant were treated with the indicated concentrations of gefitinib, erlotinib, or CL-387,785 immediately prior to suspension in soft agar. This assay was not done in triplicate, but the results are representative of two independent experiments. The number of colonies was normalized to maximum colony formation for each treatment, and sigmoidal dose response curves were fitted to the data using Prism Graphpad software to determine IC50s.

## Supporting information

S1 File Printout of Excel file from lab notebook of results of manual quantification of soft agar photos underlying charts in Fig 4B and 4C. (PDF)

S2 File Printout of Excel file from lab notebook of results of manual quantification of soft agar photos underlying charts in Fig 4B and 4C. (PDF)

S3 File Original and replicate composite soft agar photos underlying Fig 4A. Slide 2 has the erlotinib data that appears in [1] (Tarceva is the trade name for erlotinib). Iressa (gefitinib), AEE788, and CGP59326 are other EGFR inhibitors. Iressa/gefitinib works similarly to Tarceva/erlotinib. AEE788 is a multi-kinase inhibitor, including EGFR and ERBB2, and CGP59326 is an EGFR inhibitor. In this original data, the columns labeled "0.00001", "0.0001" and "100" do not appear in the final figure. Also, the row labeled "L858R+EGF" was excluded from the final figure. We abbreviate D770_N771insNPG as "Ins" in the original data, and we abbreviated L747_E749del,A750P as "del3" in the original data. NPG was the only insertion we were working with, whereas we performed experiments on multiple deletion mutants. All of these soft agar experiments were run at the same time.
(PPT)

S4 File Original blots supporting the EGFR panels in Fig 5. The top two westerns are wild-type EGFR cells treated with EGF and gefitinib (left) and CL-387,785 (right). The next two westerns below are L858R EGFR cells treated with gefitinib (left) and CL-387,785 (right). The next two westerns below are D770_N771insNPG cells treated with gefitinib (left) and CL-387,785 (right). The fourth row of two westerns and bottom row of one western are the same three cell lines treated with HKI-272. These are all anti-EGFR westerns.
(TIF)

S5 File Copy of lab notebook page with original blots underlying the phospho-EGFR Y1068 panels in Fig 5. (PDF)

S6 File Copy of lab notebook page with original blots underlying the actin panels in Fig 5. (PDF)

S7 File Copy of lab notebook page with original blots underlying the EGFR panels in Fig 5. (PDF)

S8 File Soft agar experiment of NIH-3T3 cells expressing wild-type EGFR+EGF, L858R EGFR, and exon 20 insertion EGFR (insNPG) in triplicate. For the photos, the inhibitors added from left to right are CL-387,785; erlotinib, and gefitinib when the page is upright.
(PDF)

S9 File Soft agar experiment of NIH-3T3 cells expressing wild-type EGFR+EGF, L858R EGFR, and exon 20 insertion EGFR (insNPG) in triplicate. (PDF)

S10 File Soft agar experiment comparing pooled and clonal NIH-3T3 cells expressing only insNPG EGFR. Tarceva = erlotinib, Iressa = gefitinib, CL = CL-387,785.
(PDF)

S11 File Soft agar experiment comparing pooled and clonal NIH-3T3 cells expressing only insNPG EGFR. Tarceva = erlotinib, Iressa = gefitinib, CL = CL-387,785. Quantification is notebook entry #8 for that day.
(PDF)

S12 File Soft agar experiment comparing activity of four inhibitors on NIH-3T3 cells expressing only insNPG EGFR. Iressa = gefitinib, Tarceva = erlotinib; others as labeled.
(PDF)

S13 File Soft agar experiment in triplicate comparing activity of three inhibitors on NIH-3T3 cells expressing wild-type EGFR, L858R EGFR, or exon 20 insertion EGFR (Iressa = gefitinib, Tarceva = erlotinib). ins124 = EGFR insNPG
(PDF)

S14 File Soft agar experiment in triplicate comparing activity of three inhibitors on NIH-3T3 cells expressing wild-type EGFR, L858R EGFR, or exon 20 insertion EGFR (Iressa = gefitinib, Tarceva = erlotinib). ins124 = EGFR insNPG
(PDF)

S15 File Experiment to compare activity of erlotinib (Tarceva) against soft agar colony formation in NIH-3T3 cells expressing wild-type EGFR, L858R EGFR, insNPG EGFR (ins124), and two newly identified exon 20 insertions of EGFR, insWASV and ins WH. (PDF)

S16 File Experiment to compare activity of erlotinib (Tarceva) against soft agar colony formation in NIH-3T3 cells expressing wild-type EGFR, L858R EGFR, insNPG EGFR (ins124), and two newly identified exon 20 insertions of EGFR, insWASV and ins WH. (PDF)
