# Supplementary material for: Correction: Oncogenic Transformation by Inhibitor-Sensitive and -Resistant EGFR Mutants
Source: PLoS Med. 2024 Sep 16;21(9):e1004470. doi: 10.1371/journal.pmed.1004470 (PMC11405057; doi:10.1371/journal.pmed.1004470)
Supplement: S1 File — (PDF) [file pmed.1004470.s001.pdf]

③ big CP experiments (Whei) "2/25"

	G719S Iressa small colonies	G719S Iressa big colonies	G719S Iressa total	G719S Tarceva small colonies	G719S Tarceva big colonies	G719S Tarceva total	G719S AEE small colonies	G719S AEE big colonies	G719S AEE total	G719S CGP small colonies	G719S CGP big colonies	G719S CGP total
0	39	23	**62**	4	28	**32**	7	35	**42**	8	25	**33**
0.00001	32	2	**34**	6	13	**19**	5	18	**23**	5	23	**28**
0.0001	25	4	**29**	11	22	**33**	8	29	**37**	8	24	**32**
0.001	12	12	**24**	4	16	**20**	5	29	**34**	8	19	**27**
0.01	17	1	**18**	8	4	**12**	7	4	**11**	6	11	**17**
0.1	8	0	**8**	0	1	**1**	0	1	**1**	6	14	**20**
1	2	0	**2**	0	0	**0**	0	0	**0**	3	8	**11**
10	0	0	**0**	0	0	**0**	0	0	**0**	1	0	**1**
100	0	0	**0**	0	0	**0**	0	0	**0**	0	0	**0**

	ins124 Iressa small colonies	ins124 Iressa big colonies	ins124 Iressa total	ins124 Tarceva small colonies	ins124 Tarceva big colonies	ins124 Tarceva total	ins124 AEE small colonies	ins124 AEE big colonies	ins124 AEE total	ins124 CGP small colonies	ins124 CGP big colonies	ins124 CGP total
0	13	40	**53**	24	29	**53**	14	35	**49**	22	21	**43**
0.00001	23	43	**66**	14	31	**45**	18	30	**48**	21	21	**42**
0.0001	26	34	**60**	17	26	**43**	23	32	**55**	12	19	**31**
0.001	20	40	**60**	10	20	**30**	4	31	**35**	14	21	**35**
0.01	21	36	**57**	24	26	**50**	16	29	**45**	15	22	**37**
0.1	17	39	**56**	24	29	**53**	22	31	**53**	19	26	**45**
1	15	24	**39**	14	25	**39**	8	14	**22**	19	17	**36**
10	0	0	**0**	0	0	**0**	0	0	**0**	23	21	**44**
100	0	0	**0**	0	0	**0**	0	0	**0**	4	23	**27**

	del3 Iressa small colonies	del3 Iressa big colonies	del3 Iressa total	del3 Tarceva small colonies	del3 Tarceva big colonies	del3 Tarceva total	del3 AEE small colonies	del3 AEE big colonies	del3 AEE total	del3 CGP small colonies	del3 CGP big colonies	del3 CGP total
0	28	24	**52**	24	16	**40**	24	15	**39**	33	7	**40**
0.00001	28	5	**33**	27	14	**41**	30	7	**37**	20	7	**27**
0.0001	10	0	**10**	22	5	**27**	14	18	**32**	25	16	**41**
0.001	27	5	**32**	30	14	**44**	29	13	**42**	22	10	**32**
0.01	12	0	**12**	18	15	**33**	7	7	**14**	29	10	**39**
0.1	0	0	**0**	0	0	**0**	0	0	**0**	29	13	**42**
1	0	0	**0**	0	0	**0**	0	0	**0**	21	11	**32**
10	0	0	**0**	0	0	**0**	0	0	**0**	0	0	**0**
100	0	0	**0**	0	0	**0**	0	0	**0**	0	0	**0**

see 3/27 #1 for graphs
[illegible]
see 2/20 #1 for transitions
