# Supplementary material for: Correction: Oncogenic Transformation by Inhibitor-Sensitive and -Resistant EGFR Mutants
Source: PLoS Med. 2024 Sep 16;21(9):e1004470. doi: 10.1371/journal.pmed.1004470 (PMC11405057; doi:10.1371/journal.pmed.1004470)
Supplement: S2 File — (PDF) [file pmed.1004470.s002.pdf]

2-25-05 Soft Agar Colony Formation

	WT + EGF Iressa			WT + EGF Tarceva			WT + EGF AEE			WT + EGF CGP		
	small colonies	big colonies	total	small colonies	big colonies	total	small colonies	big colonies	total	small colonies	big colonies	total
0	12	17	29	19	29	48	19	30	49	20	15	35
0.00001	14	22	36	30	28	58	13	0	13	19	8	27
0.0001	20	27	47	14	22	36	16	16	32	20	15	35
0.001	8	21	29	15	16	31	16	15	31	7	16	23
0.01	25	16	41	36	17	53	17	19	36	10	14	24
0.1	21	10	31	14	15	29	21	17	38	15	13	28
1	12	4	16	10	5	15	0	0	0	4	15	19
10	0	0	0	0	0	0	0	0	0	15	4	19
100	0	0	0	0	0	0	0	0	0	0	0	0

	L858R Iressa			L858R Tarceva			L858R AEE			L858R CGP		
	small colonies	big colonies	total	small colonies	big colonies	total	small colonies	big colonies	total	small colonies	big colonies	total
0	23	50	73	11	19	30	5	19	24	12	22	34
0.00001	22	4	26	6	10	16	6	15	21	11	15	26
0.0001	19	0	19	6	14	20	11	23	34	8	32	40
0.001	6	11	17	8	14	22	11	25	36	9	32	41
0.01	13	0	13	2	16	18	2	3	5	14	23	37
0.1	1	0	1	1	0	1	0	0	0	8	29	37
1	0	0	0	0	0	0	0	0	0	6	19	25
10	0	0	0	0	0	0	0	0	0	0	0	0
100	0	0	0	0	0	0	0	0	0	0	0	0

	L858R + EGF Iressa			L858R + EGF Tarceva			L858R + EGF AEE			L858R + EGF CGP		
	small colonies	big colonies	total	small colonies	big colonies	total	small colonies	big colonies	total	small colonies	big colonies	total
0	27	18	45	22	13	35	30	16	46	20	16	36
0.00001	23	14	37	17	14	31	14	17	31	24	13	37
0.0001	20	6	26	20	13	33	18	14	32	35	13	48
0.001	34	16	50	10	26	36	12	23	35	16	17	33
0.01	27	8	35	16	20	36	21	26	47	15	8	23
0.1	20	0	20	2	1	3	0	3	3	21	10	31
1	0	0	0	0	0	0	0	0	0	8	18	26
10	0	0	0	0	0	0	0	0	0	0	0	0
100	0	0	0	0	0	0	0	0	0	0	0	0

[illegible]
