# Supplementary material for: Correction: Oncogenic Transformation by Inhibitor-Sensitive and -Resistant EGFR Mutants
Source: PLoS Med. 2024 Sep 16;21(9):e1004470. doi: 10.1371/journal.pmed.1004470 (PMC11405057; doi:10.1371/journal.pmed.1004470)
Supplement: S3 File — Slide 2 has the erlotinib data that appears in [1] (Tarceva is the trade name for erlotinib). Iressa (gefitinib), AEE788, and CGP59326 are other EGFR inhibitors. Iressa/gefitinib works similarly to Tarceva/erlotinib. AEE788 is a multi-kinase inhibitor, including EGFR and ERBB2, and CGP59326 is an EGFR inhibitor. In this original data, the columns labeled "0.00001", "0.0001" and "100" do not appear in the final figure. Also, the row labeled "L858R+EGF" was excluded from the final figure. We abbreviate D770_N771insNPG as "Ins" in the original data, and we abbreviated L747_E749del,A750P as "del3" in the original data. NPG was the only insertion we were working with, whereas we performed experiments on multiple deletion mutants. All of these soft agar experiments were run at the same time. (PPT) [file pmed.1004470.s003.ppt]

## Slide 1
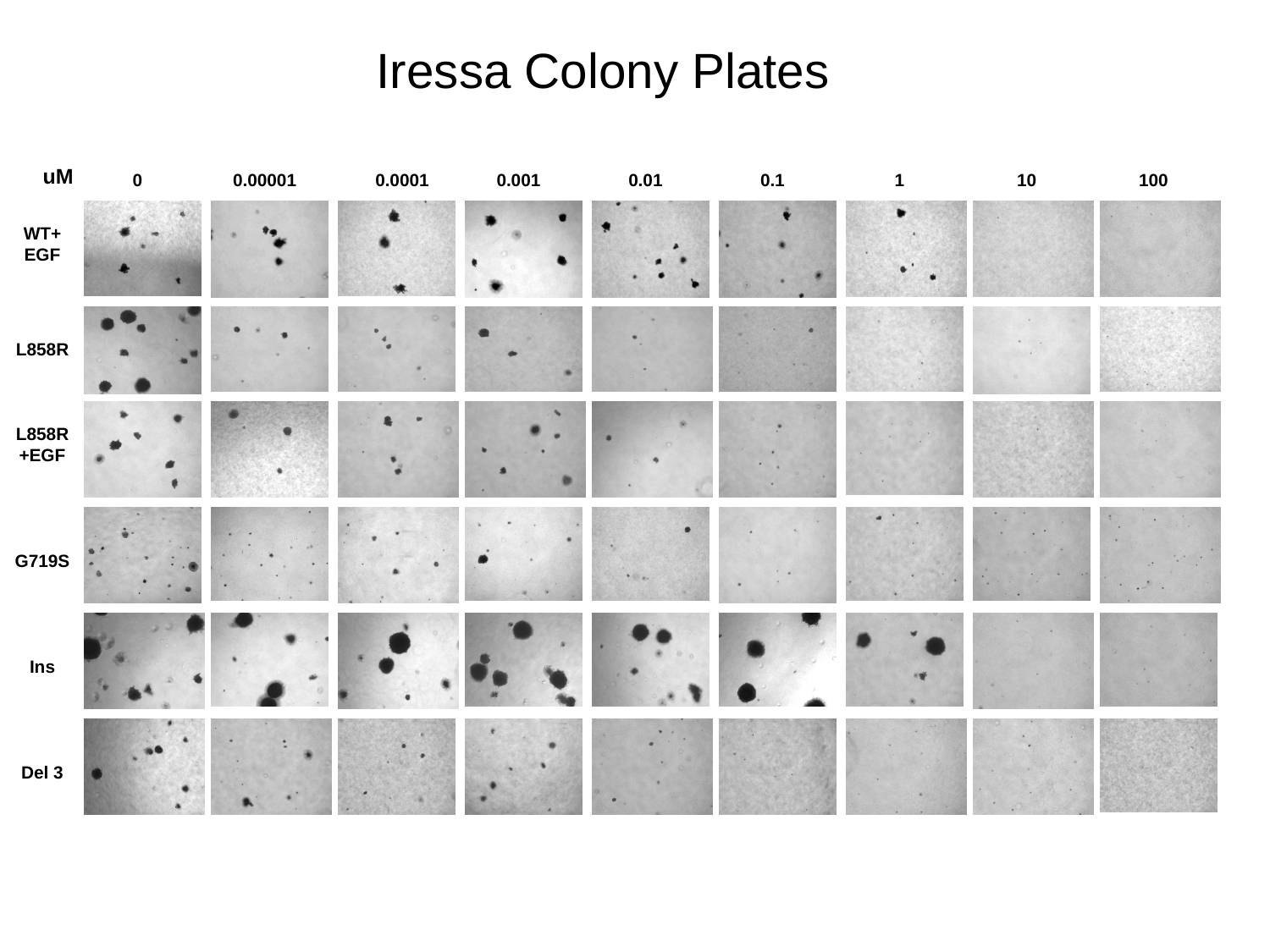

# Iressa Colony Plates
| uM |
| --- |
0
0.00001
0.0001
0.001
0.01
0.1
1
10
100
WT+ EGF
L858R
L858R+EGF
G719S
Ins
Del 3

## Slide 2
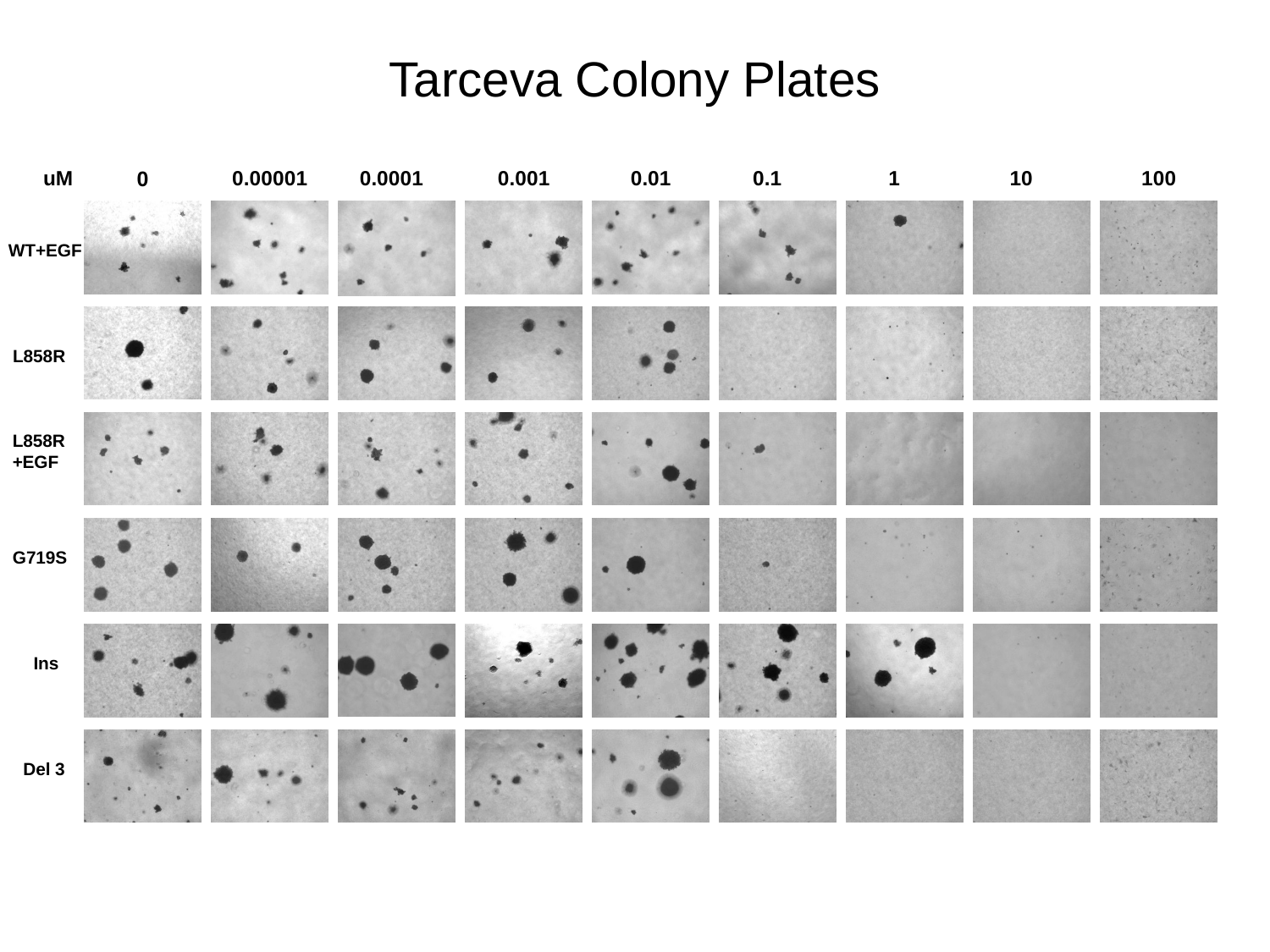

# Tarceva Colony Plates
uM
0
0.00001
0.0001
0.001
0.01
0.1
1
10
100
WT+EGF
L858R
L858R +EGF
G719S
Ins
Del 3

## Slide 3
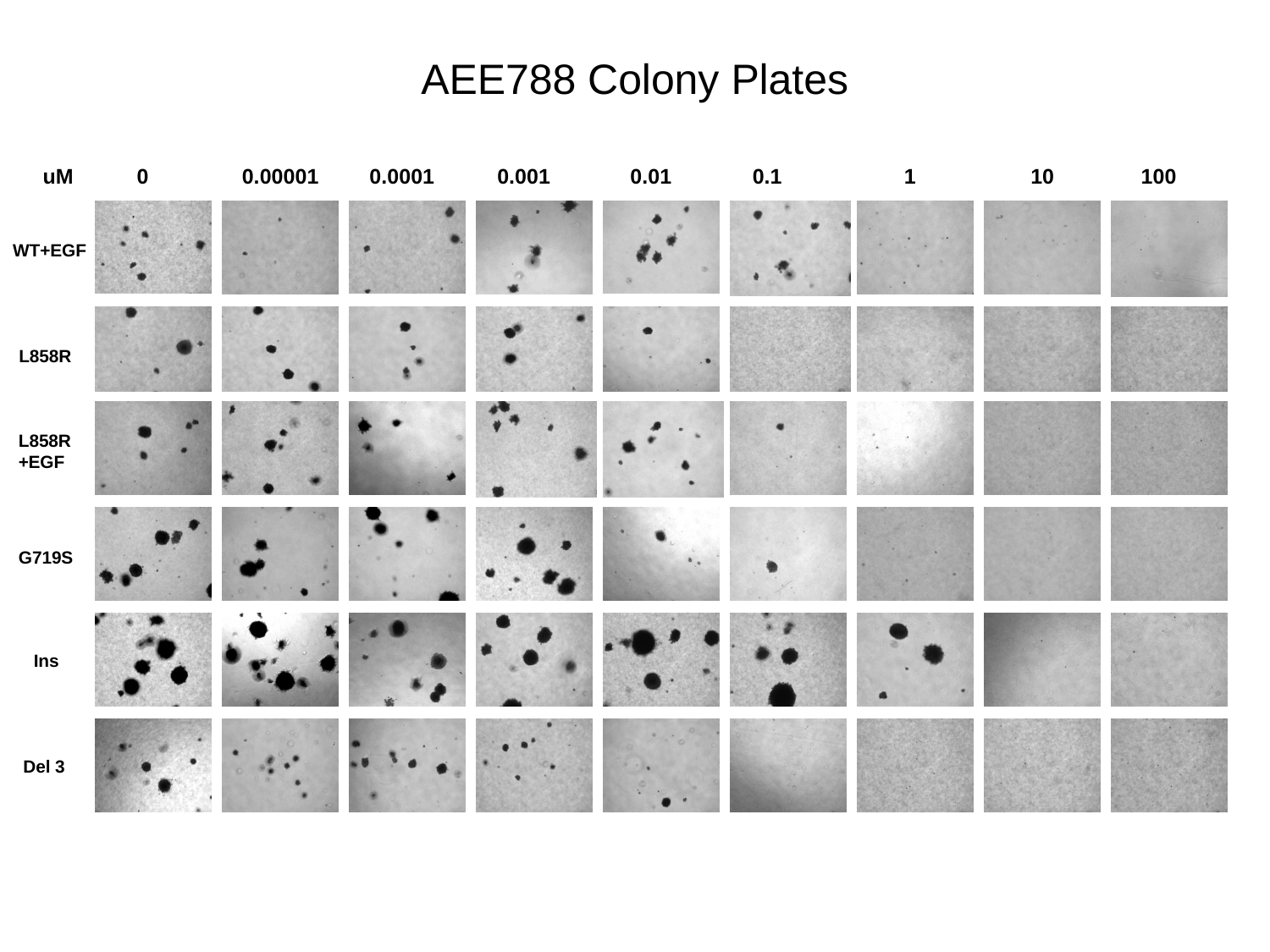

AEE788 Colony Plates
uM
| 0 |
| --- |
| 0.00001 |
| --- |
| 0.0001 |
| --- |
| 0.001 |
| --- |
| 0.01 |
| --- |
| 0.1 |
| --- |
| 1 |
| --- |
| 10 |
| --- |
| 100 |
| --- |
WT+EGF
L858R
L858R +EGF
G719S
Ins
Del 3

## Slide 4
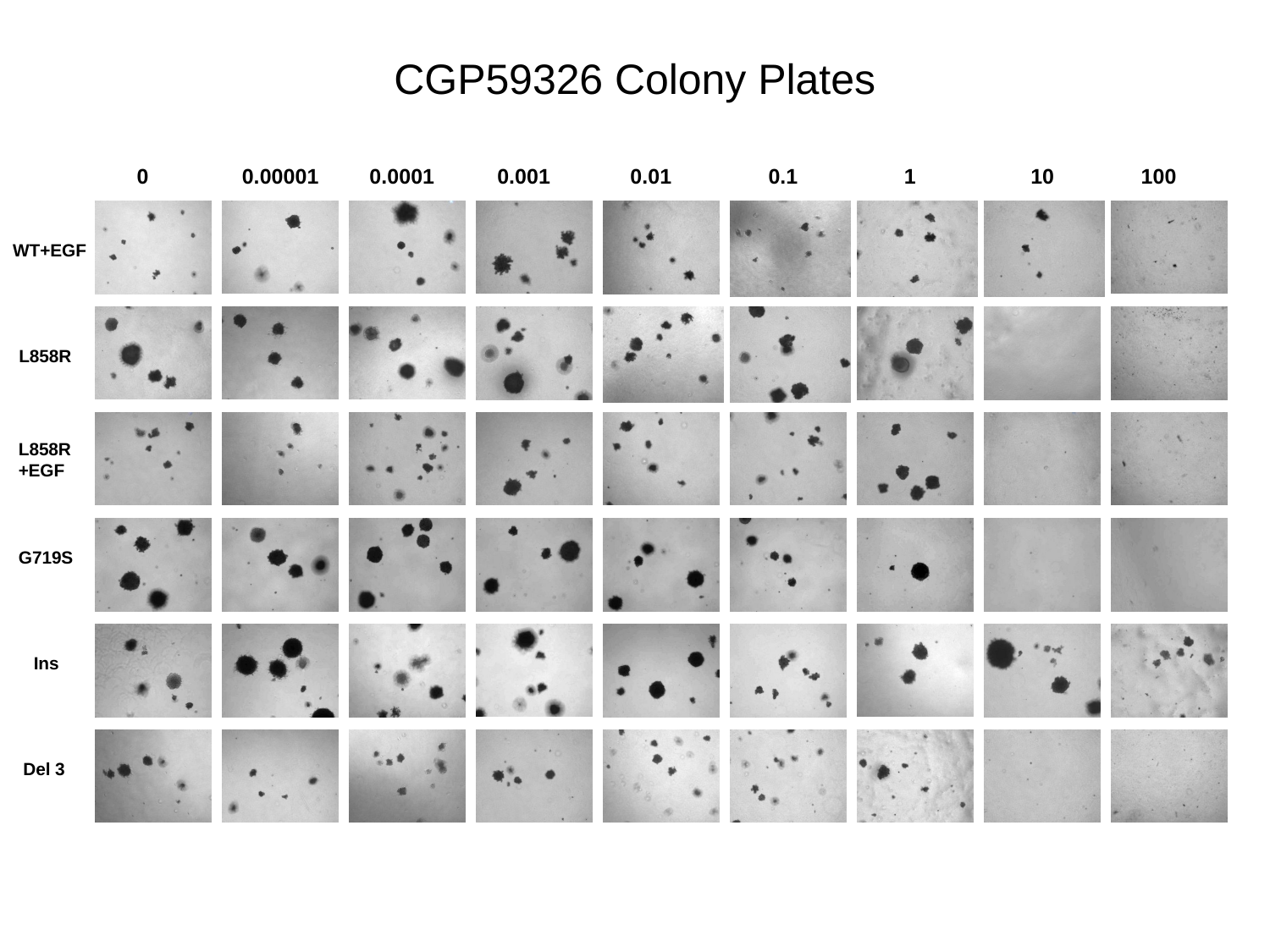

CGP59326 Colony Plates
| 0 |
| --- |
| 0.00001 |
| --- |
| 0.0001 |
| --- |
| 0.001 |
| --- |
| 0.01 |
| --- |
| 0.1 |
| --- |
| 1 |
| --- |
| 10 |
| --- |
| 100 |
| --- |
WT+EGF
L858R
L858R +EGF
G719S
Ins
Del 3
