# Supplementary material for: Correction: Oncogenic Transformation by Inhibitor-Sensitive and -Resistant EGFR Mutants
Source: PLoS Med. 2024 Sep 16;21(9):e1004470. doi: 10.1371/journal.pmed.1004470 (PMC11405057; doi:10.1371/journal.pmed.1004470)
Supplement: S5 File — (PDF) [file pmed.1004470.s005.pdf]

③ [illegible] Iressa/CL [illegible] curve α ⓅEGFR Y1068 W

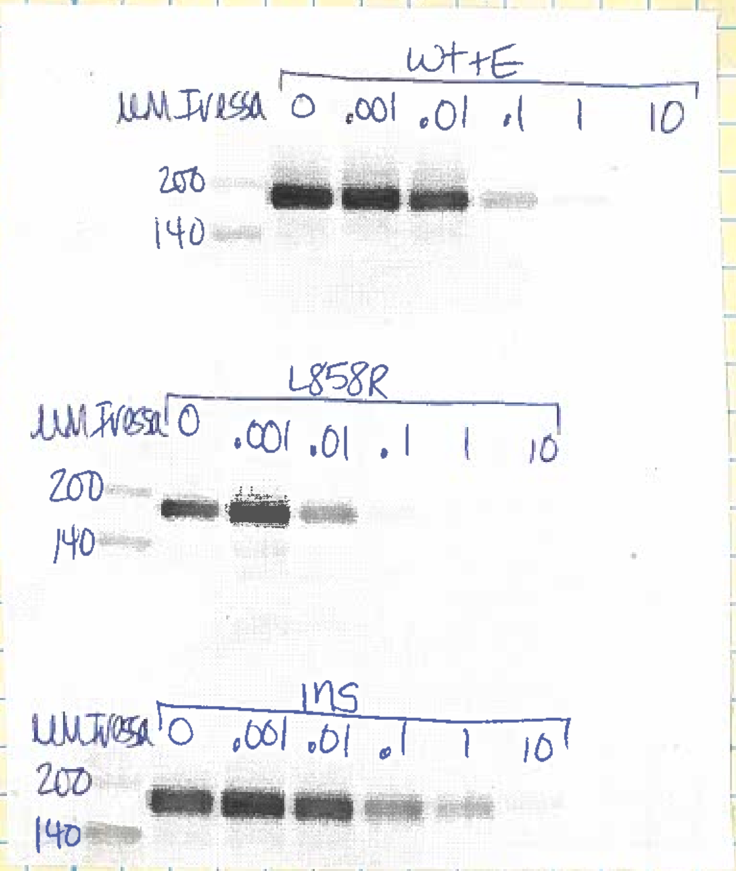

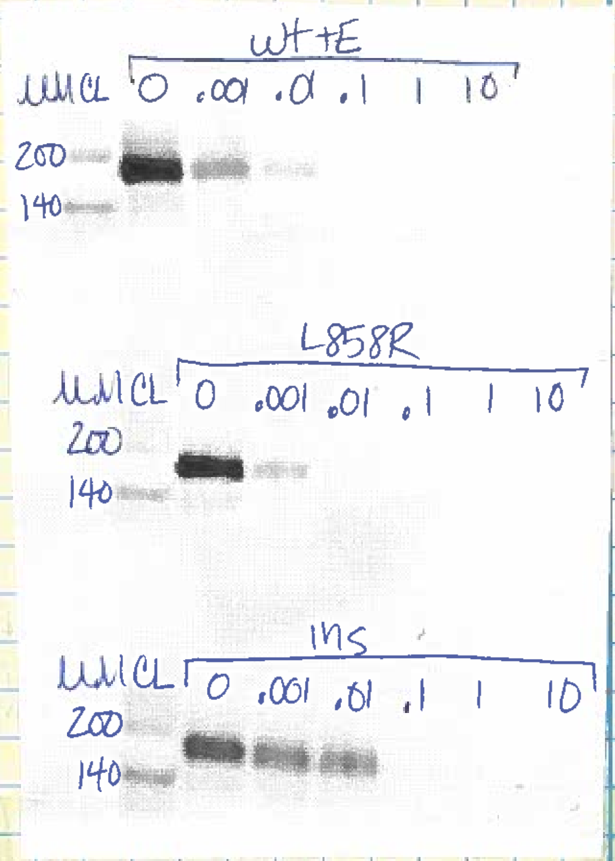

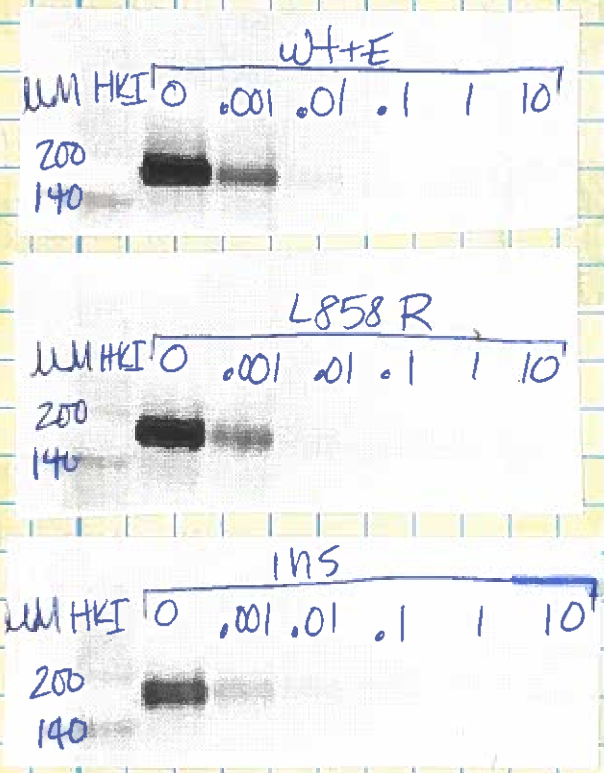

looks great
wt+E is clearly less sensitive to
Iressa than L858R, & ins is [illegible]
less sensitive than that
CL is 10x more effective than [illegible]
for wt+E & L858R, & 100x more
effective than Iressa for [illegible]
HKI appears to be equally effective
for all 3 cell types
these results more consistent w/
colony data than 5/26 #3
these results roughly correlate
with 5/4 #5 & 5/4 #8 colony [illegible]
for Iressa & CL ± 1 log
HKI is extremely effective for [illegible]
of EGFR auto Ⓟ ation but [illegible]
doesn't correlate w/ colony data
(see 6/25 #1) - cpd stability [illegible]
over 3 wk assay?
see ④ for anti-actin
