# Supplementary material for: Correction: Oncogenic Transformation by Inhibitor-Sensitive and -Resistant EGFR Mutants
Source: PLoS Med. 2024 Sep 16;21(9):e1004470. doi: 10.1371/journal.pmed.1004470 (PMC11405057; doi:10.1371/journal.pmed.1004470)
Supplement: S6 File — (PDF) [file pmed.1004470.s006.pdf]

④ Western anti-actin W/ for αⓅEGFR (see ③)

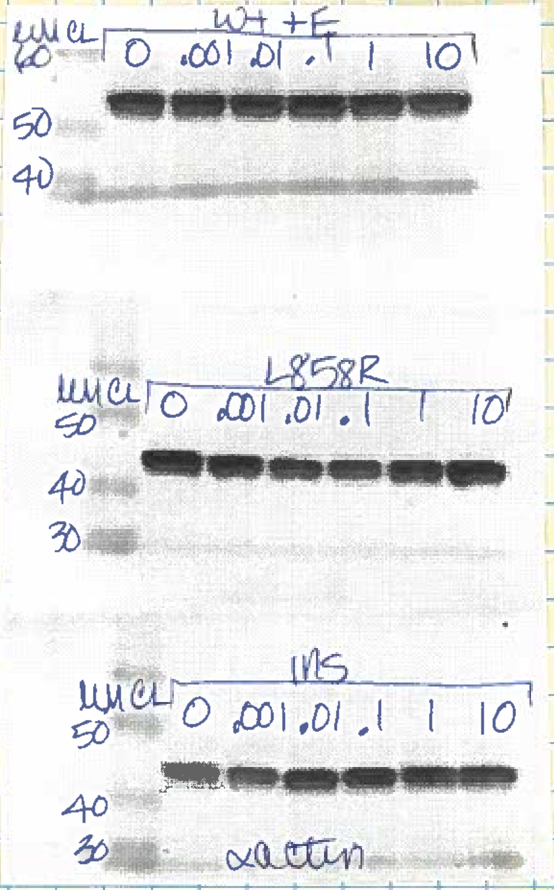

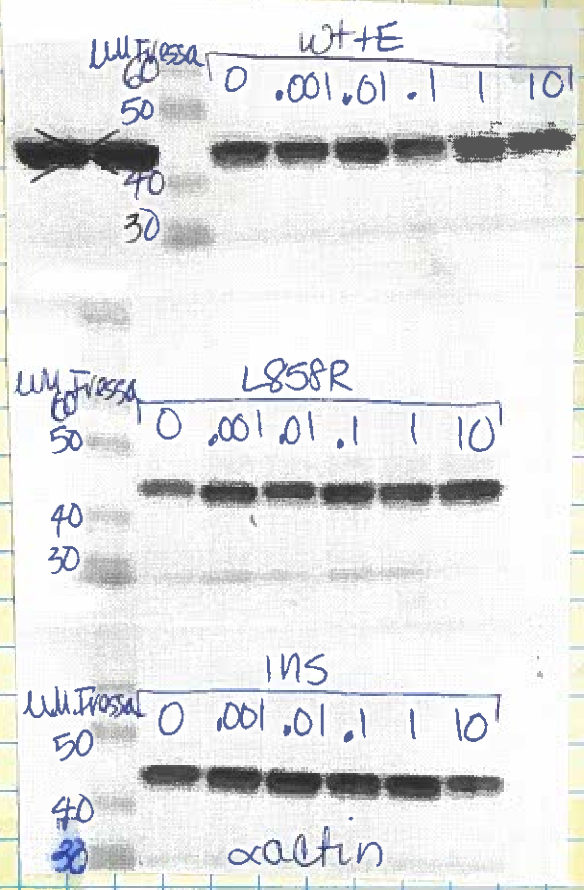

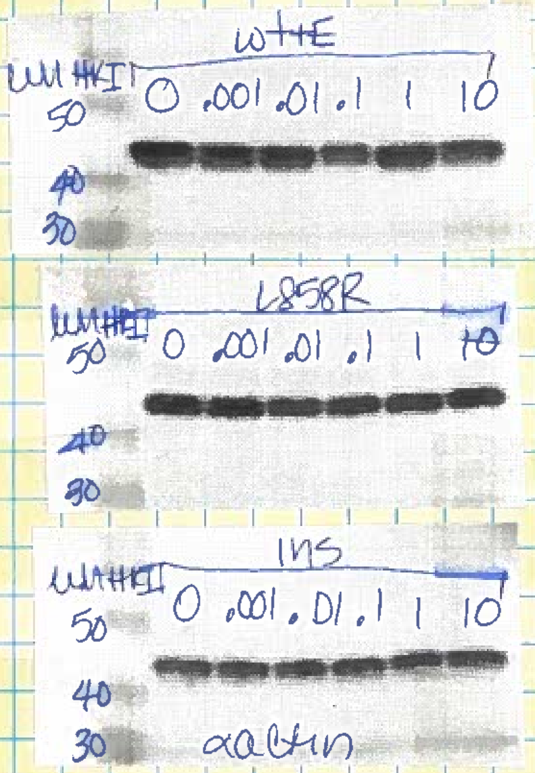

loading is equal
see ③ for αⓅEGFR Y1068
see 7/18 #1 for αEGFR
