# Supplementary material for: Correction: Oncogenic Transformation by Inhibitor-Sensitive and -Resistant EGFR Mutants
Source: PLoS Med. 2024 Sep 16;21(9):e1004470. doi: 10.1371/journal.pmed.1004470 (PMC11405057; doi:10.1371/journal.pmed.1004470)
Supplement: S7 File — (PDF) [file pmed.1004470.s007.pdf]

7/8/06

① Whus αEGFR WB for drug dose response

see 7/6 #3 for ⓟEGFR Y1068

see 7/6 #4 for α actin

EGFR levels all the same; some of ins/CL sample may have floated out of well

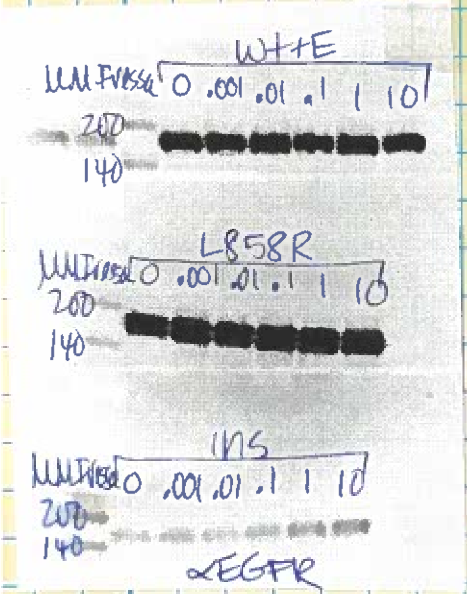

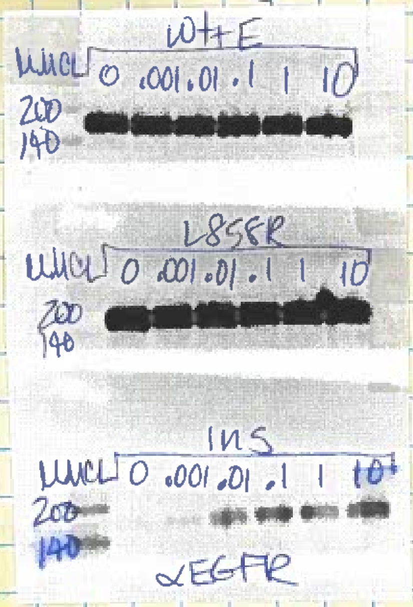

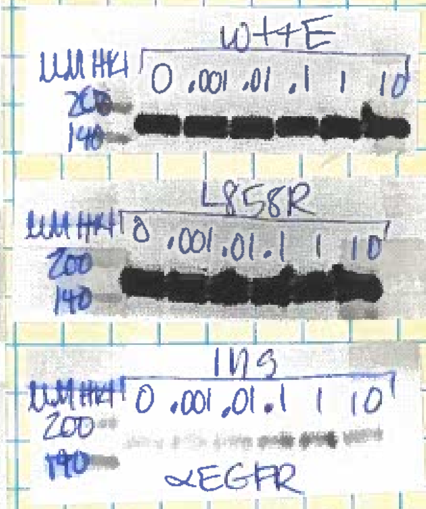

see 7/20 #2 for ins+CL repeat

7/12/05

① Pasi's summary of ins mutation patients w/ treatment

	Reference		Response to treaten	Reference	Agent	Not
**eated patients**		**Treated patients**				
V770-772 ins	Shigematsu JNCI 2005	S768-V769 SVAins	Stable disease	Eberhard et al	Tarceva	w/ch
H774 ins	Shigematsu JNCI 2005	M766-A767 AIins	Stable disease	Eberhard et al	Tarceva	w/ch
G771 ins	Shigematsu JNCI 2005	D770_N771 ins SVD	Stable disease	Sequist and Lycnch	Tarceva	unpublished
'770-771 ins	Shigematsu JNCI 2005					
;-774ins, H775Y	Shigematsu JNCI 2005					
I774-775 ins	Shigematsu JNCI 2005					
H774-776 ins	Shigematsu JNCI 2005					
'775-776 ins	Shigematsu JNCI 2005					
_762insEAFQ	Huang CCR 2004					
58_D770dup	Huang CCR 2004					
_762insEAFQ	Kosaka CR 2004					
'_S768insTLA	Kosaka CR 2004					
_D770insASV	Kosaka CR 2004					
