# Supplementary figures and images for: Correction: Oncogenic Transformation by Inhibitor-Sensitive and -Resistant EGFR Mutants
Source: PLoS Med. 2024 Sep 16;21(9):e1004470. doi: 10.1371/journal.pmed.1004470 (PMC11405057; doi:10.1371/journal.pmed.1004470)

composite photo (TH) from 041505 triplicate CP  
 see ⑤ for quantitation  
 file called "ITC triplicate CP  
 050405 TH"

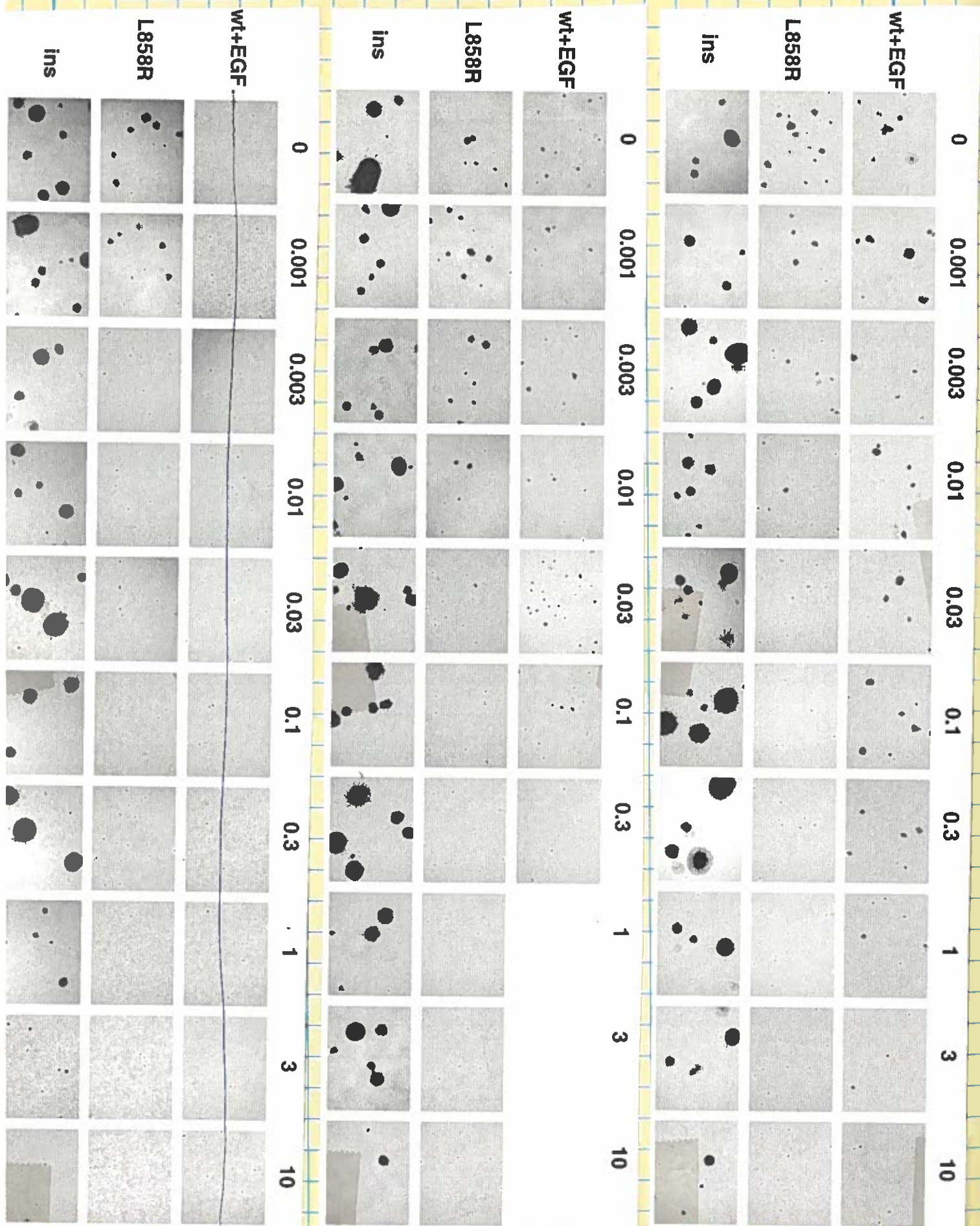

Supplement: S8 File — For the photos, the inhibitors added from left to right are CL-387,785; erlotinib, and gefitinib when the page is upright. (PDF) [file pmed.1004470.s008.pdf]

① This 062205 was CP  
 HIV-2 cannot seem to be affected in this assay  
 to HIV-1 degraded over time

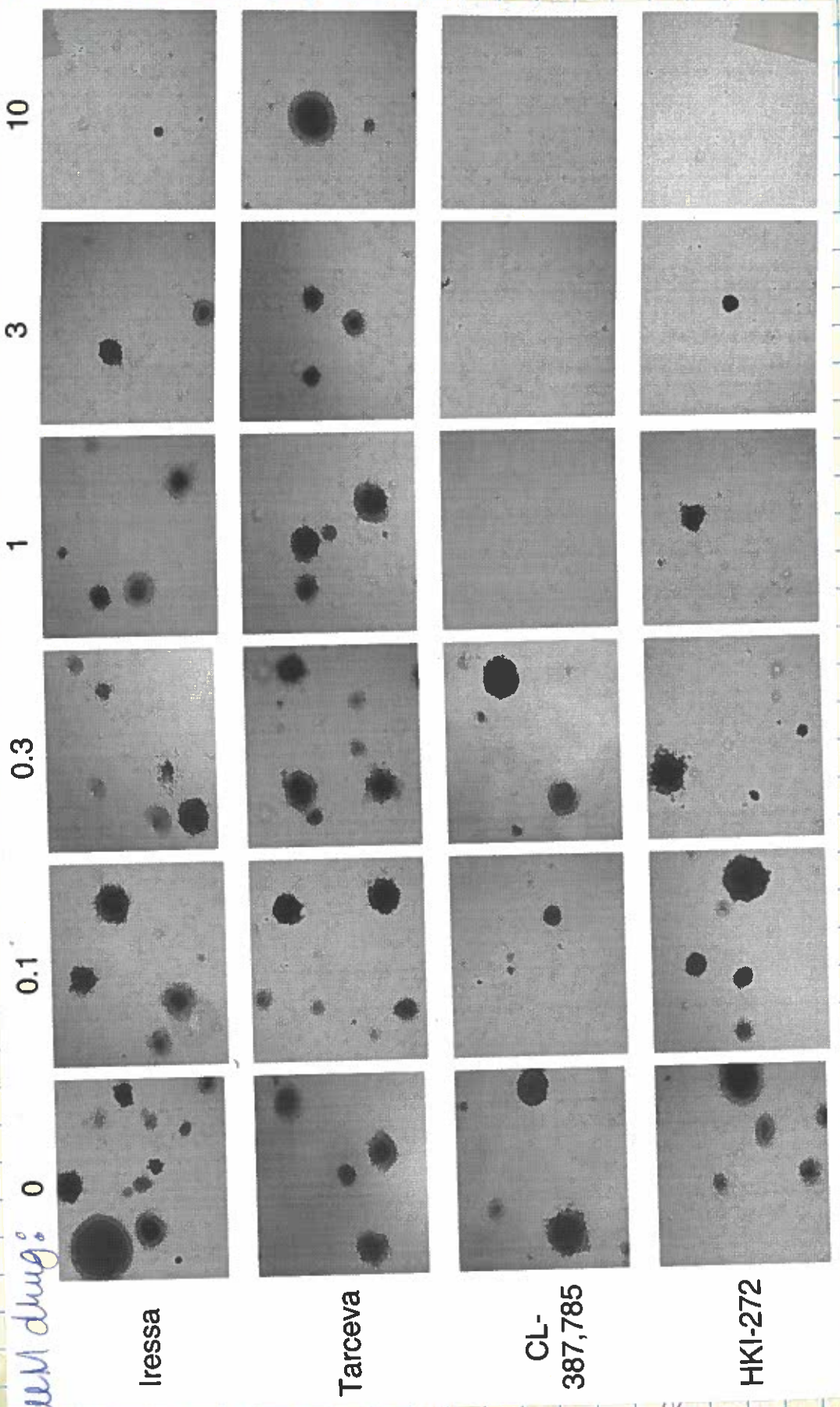

062205 HIV-2

Supplement: S12 File — Iressa = gefitinib, Tarceva = erlotinib; others as labeled. (PDF) [file pmed.1004470.s012.pdf]

9/1/05

DTG's colony assay w/ full panel of EGFR ins & del m

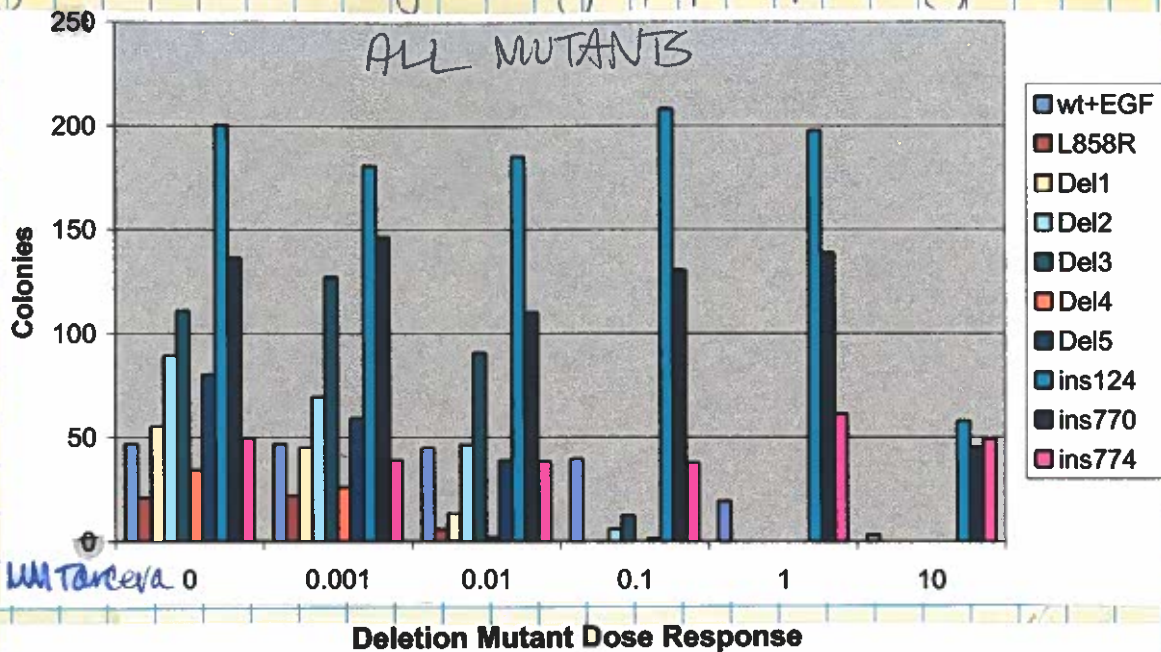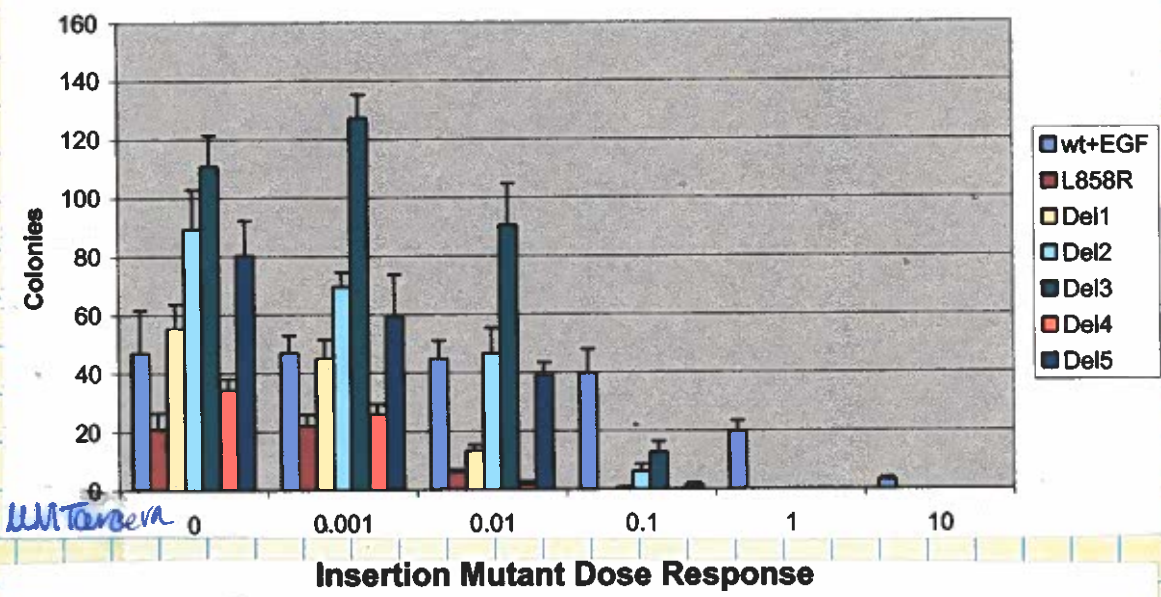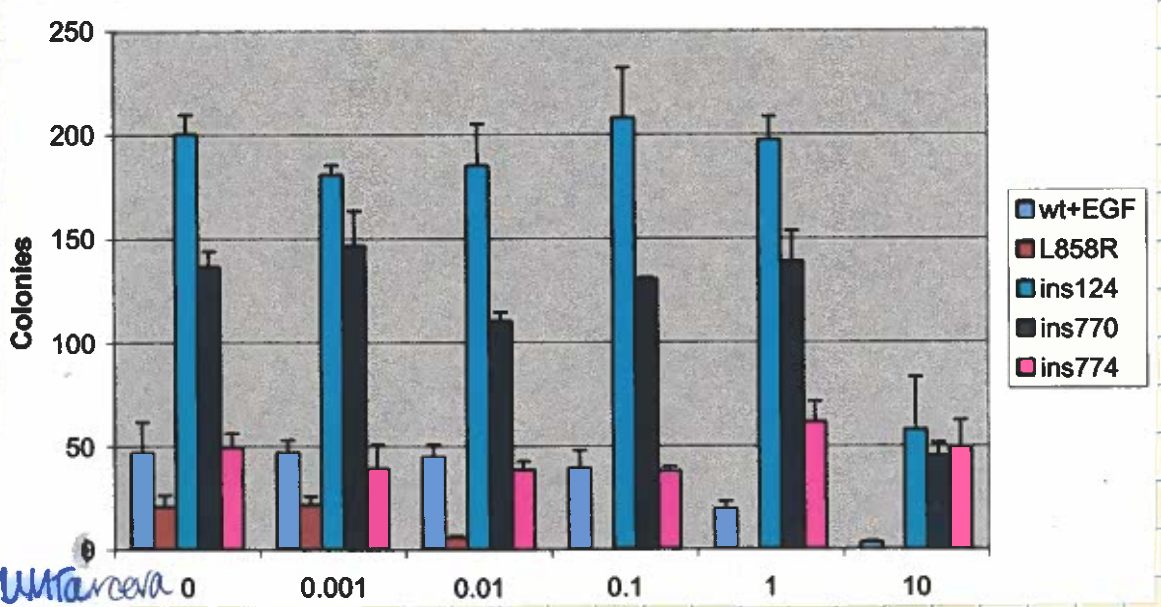

DTG ins NPG

770 = 9LDT10VETV

774 = 73\_VT4insH

see 8/10/05

see 10/10/05

Supplement: S16 File — (PDF) [file pmed.1004470.s016.pdf]
