# Supplementary material for: Correction: Oncogenic Transformation by Inhibitor-Sensitive and -Resistant EGFR Mutants
Source: PLoS Med. 2024 Sep 16;21(9):e1004470. doi: 10.1371/journal.pmed.1004470 (PMC11405057; doi:10.1371/journal.pmed.1004470)
Supplement: S9 File — (PDF) [file pmed.1004470.s009.pdf]

⑤ graphs from [illegible] triplicate colony experiment

"041505 Ires Tarceva CL

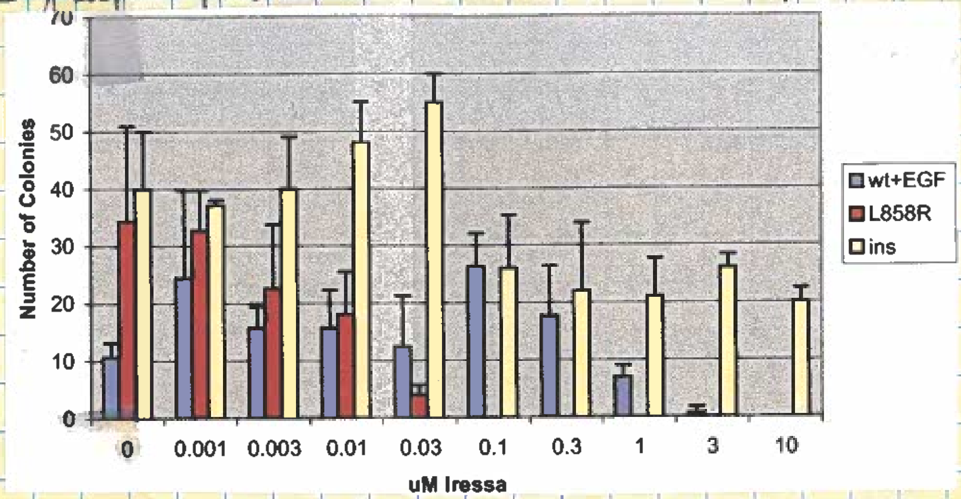

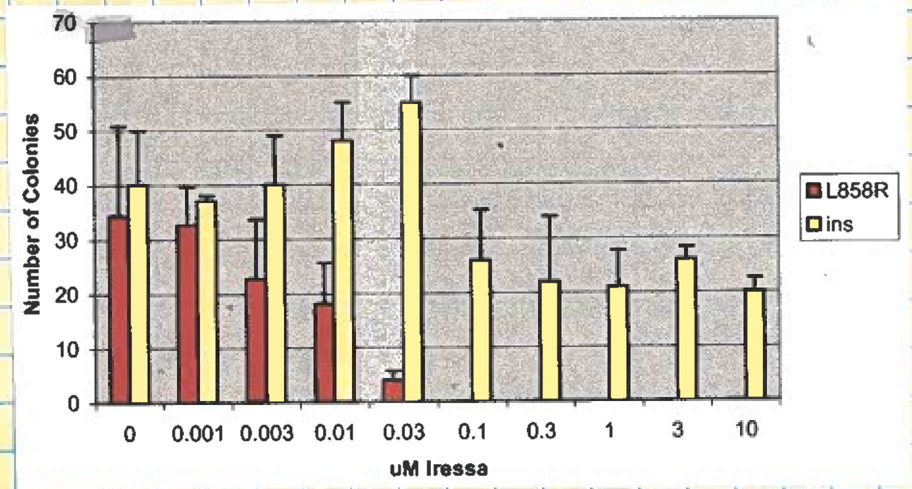

missing wt+EGF data for Tarceva

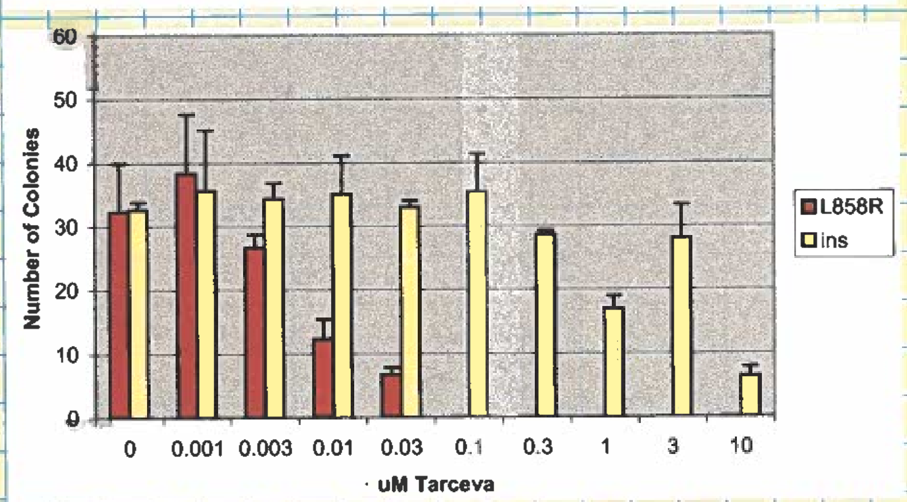

EGF not added wt for CL

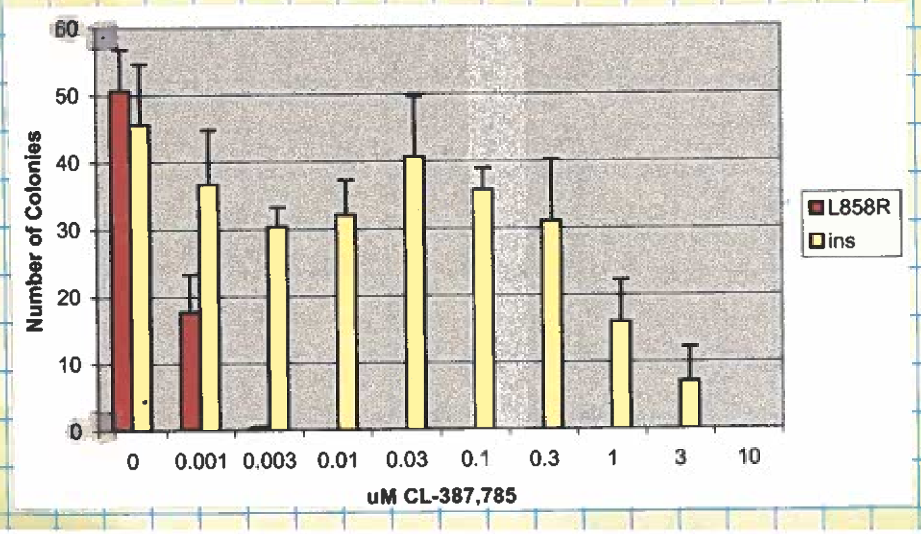

see ⑥ for [illegible]
