# Supplementary material for: Correction: Oncogenic Transformation by Inhibitor-Sensitive and -Resistant EGFR Mutants
Source: PLoS Med. 2024 Sep 16;21(9):e1004470. doi: 10.1371/journal.pmed.1004470 (PMC11405057; doi:10.1371/journal.pmed.1004470)

5/6/05

① This photo composite for insertion mutant pool/polyclonal data indicates no mutations that accumulated during isolation of clonal cell line are responsible for ins mutant resistance to Iressa/Tarceva

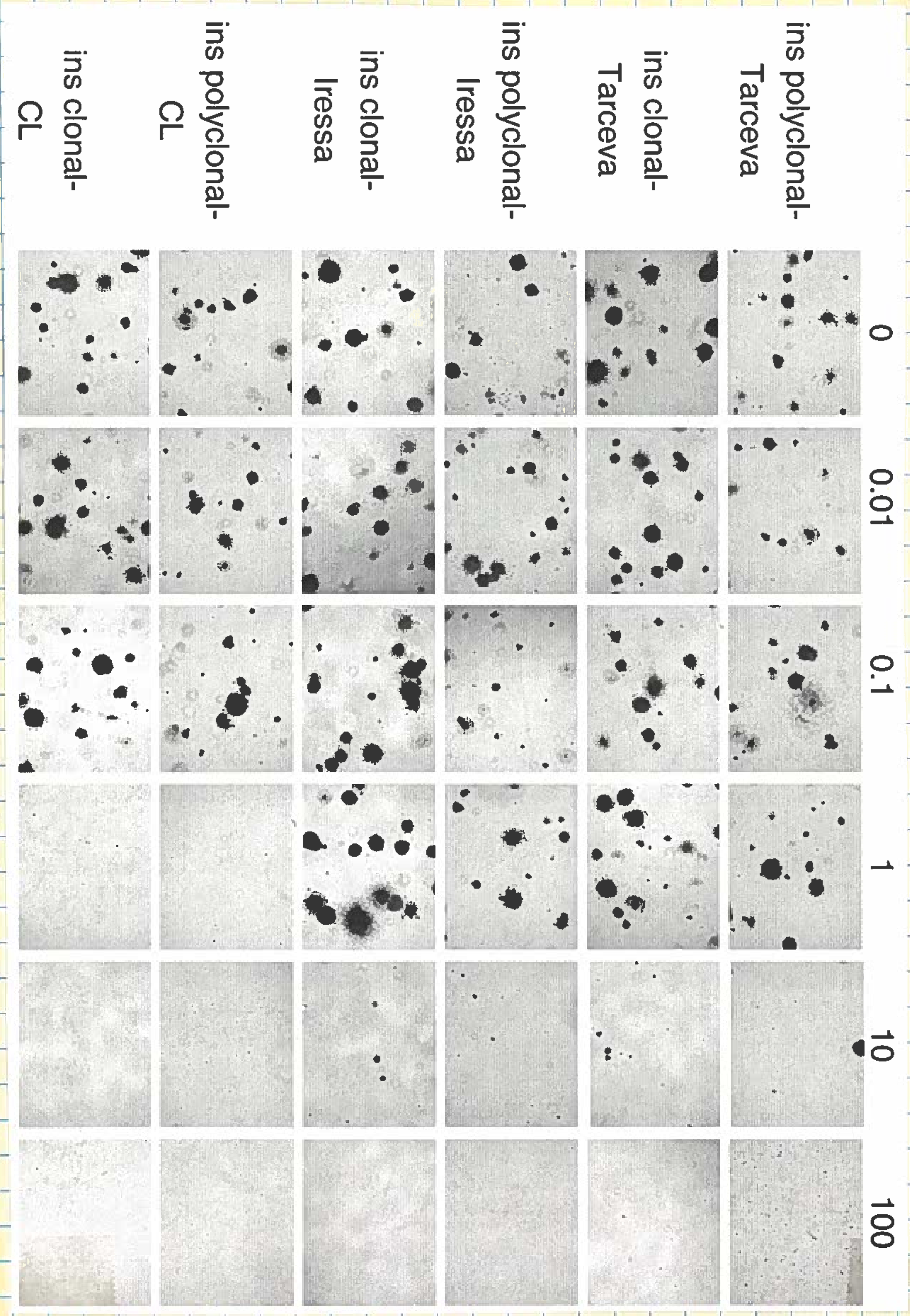

Supplement: S10 File — Tarceva = erlotinib, Iressa = gefitinib, CL = CL-387,785. (PDF) [file pmed.1004470.s010.pdf]
