# Supplementary material for: Correction: Oncogenic Transformation by Inhibitor-Sensitive and -Resistant EGFR Mutants
Source: PLoS Med. 2024 Sep 16;21(9):e1004470. doi: 10.1371/journal.pmed.1004470 (PMC11405057; doi:10.1371/journal.pmed.1004470)
Supplement: S11 File — Tarceva = erlotinib, Iressa = gefitinib, CL = CL-387,785. Quantification is notebook entry #8 for that day. (PDF) [file pmed.1004470.s011.pdf]

(7) TH's anti-EGFR WB on cells from CP w/ Yukie's double mutant
see ④ for preliminary CP data summary
see 3/11 #1 for abbreviations

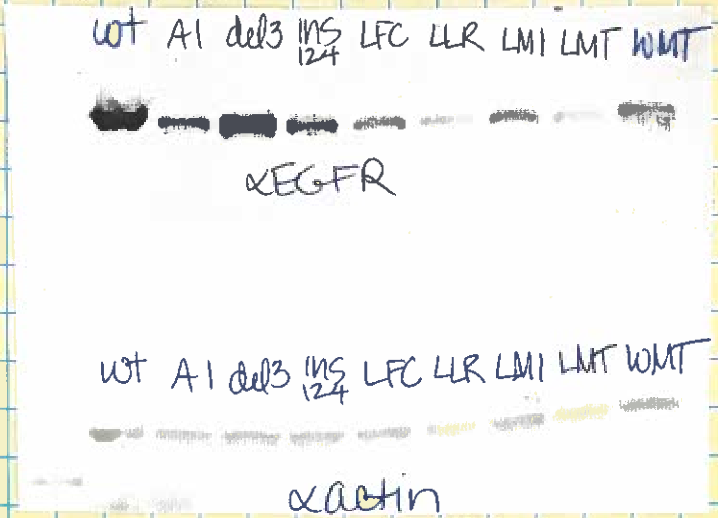

Yukie's mutants selected puro 16 days; ctrl b10c[illegible]

see 5/16 #1 for CP photos

(8) TH's pooled stable vs clonal insertion mut CP

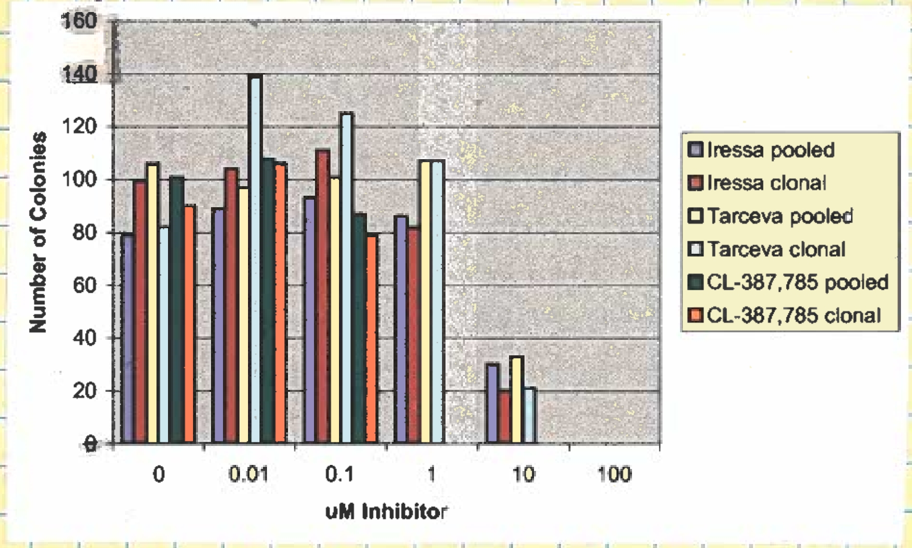

"TH 050405 pooled [illegible]
clonal [illegible] quan[illegible]

CL more effective this experiment in ⑤ triplicate exp

see photos (5/6 #1) explain discrepan[illegible] a few small colonies not counted here were counted in triplicate exp (5

(9) Prism graph of clonal data normalized to maximal #colon[illegible]

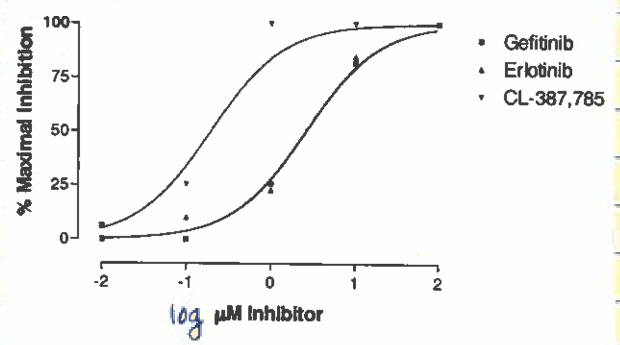

	Gefitinib	Erlotinib	CL-387,785
BOTTOM (Constant)	0.0	0.0	0.0
TOP (Constant)	100.0	100.0	100.0
LOGEC50	0.4122	0.4016	-0.7172
EC50	2.583	2.521	0.1918
