# Supplementary material for: Correction: Oncogenic Transformation by Inhibitor-Sensitive and -Resistant EGFR Mutants
Source: PLoS Med. 2024 Sep 16;21(9):e1004470. doi: 10.1371/journal.pmed.1004470 (PMC11405057; doi:10.1371/journal.pmed.1004470)

③ 070705CP: WF/TH/HG wt/AY/ins, I/T/HKI in triplicate
compound refeeding has a profound effect on colony forma[illegible]
HKI now seems to work better against ins; Iressa & Tarceva
③ for attrition — work better against wt & L858R (but not ins); correlates [illegible]
7/16 #3 & ① EGFR Y1068 data; but if cpd not degraded, then increasing [illegible]

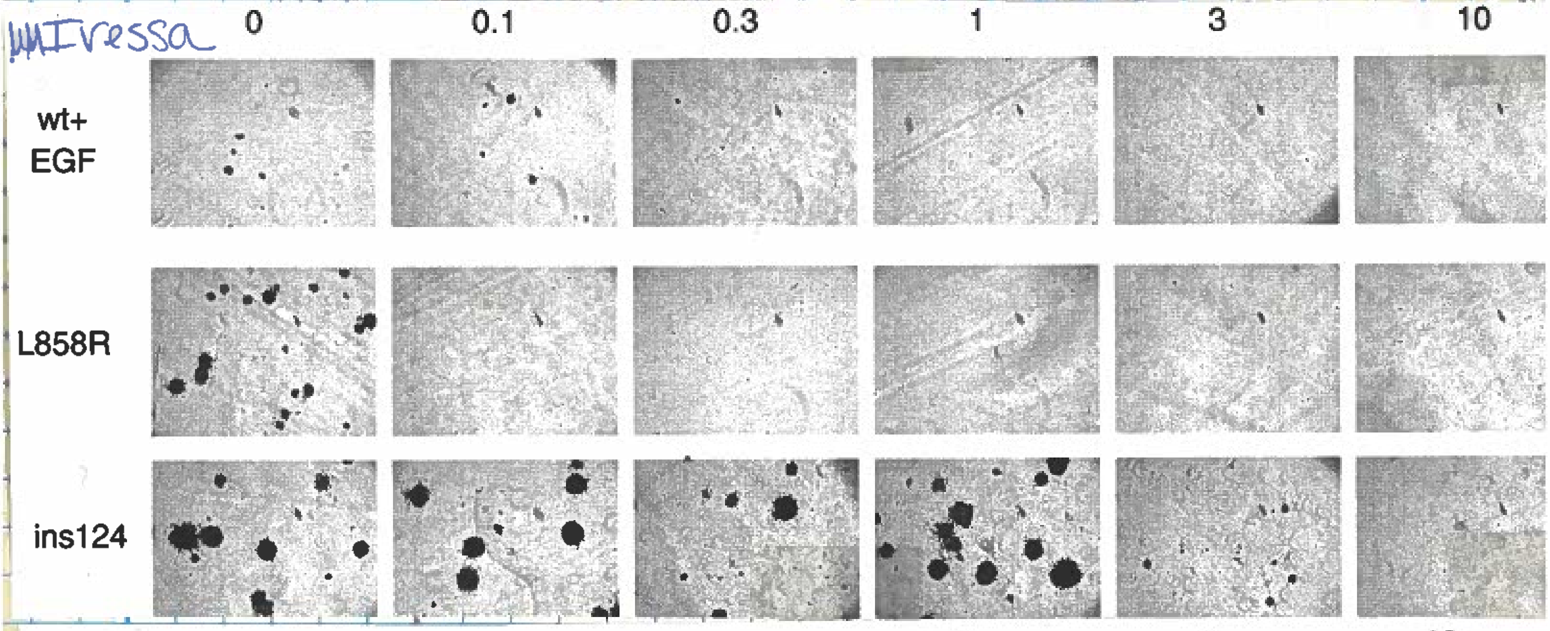

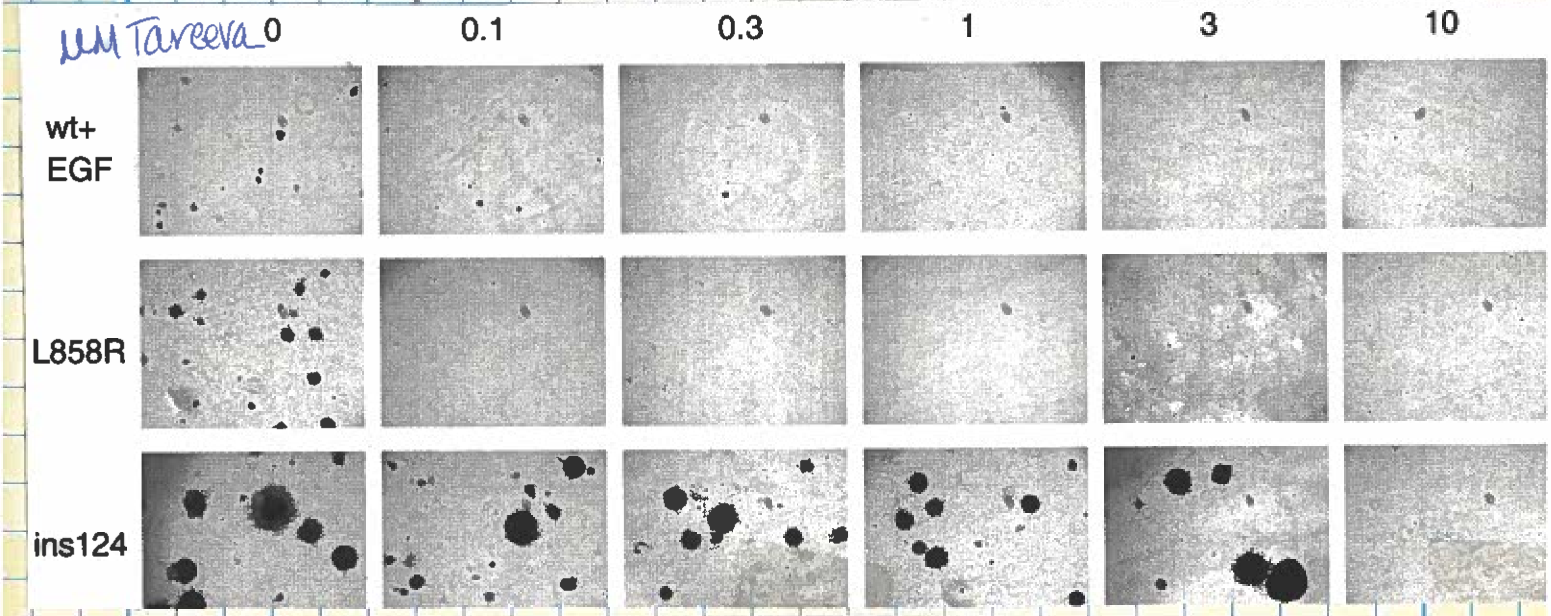

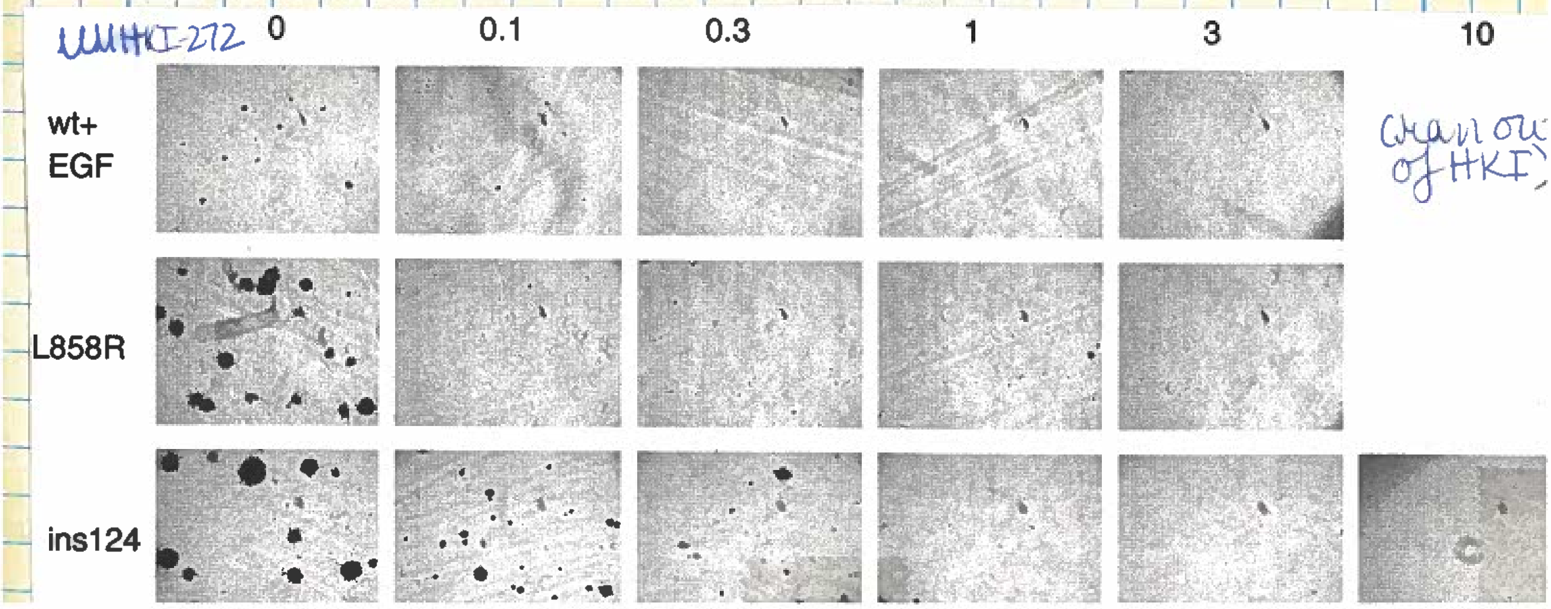

Supplement: S13 File — ins124 = EGFR insNPG (PDF) [file pmed.1004470.s013.pdf]
