# Supplementary material for: Correction: Oncogenic Transformation by Inhibitor-Sensitive and -Resistant EGFR Mutants
Source: PLoS Med. 2024 Sep 16;21(9):e1004470. doi: 10.1371/journal.pmed.1004470 (PMC11405057; doi:10.1371/journal.pmed.1004470)

(4) TH's pUSE del5HS construct
"pUSE del5HS D,E,F,G 071805"
see 3/16/05#2 for HS deletion description
2239-2250del, A2251C L747_A750del, T751P ✓
2349 (2361) CAG→CAA gln→gln (silent)
looks good!
now cut out & transfer to pBabe puro

(5) WF/TH/HG 070705/071805 CP w/ refeeding
all drug responses shifted back ~1 log but no real rel[illegible]
differences

see (3) for photos

compare w/:
10/18#2
3/18#3
5/4/05#8
6/25/05#1

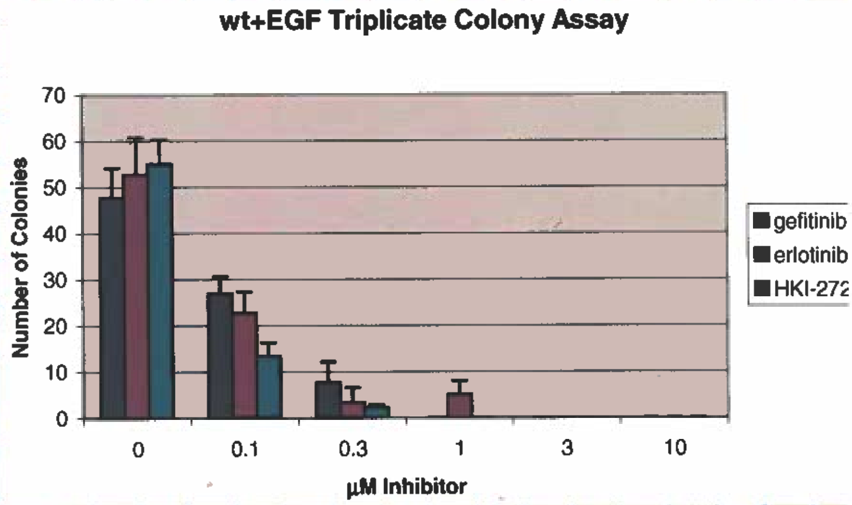

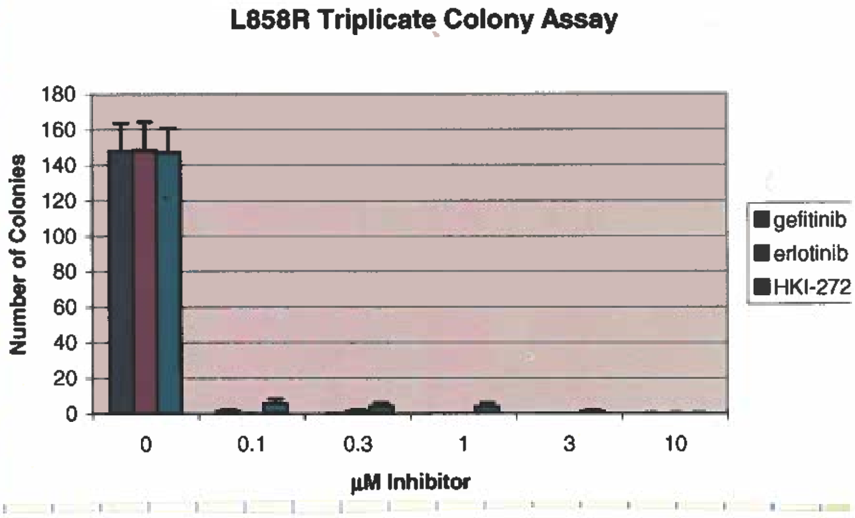

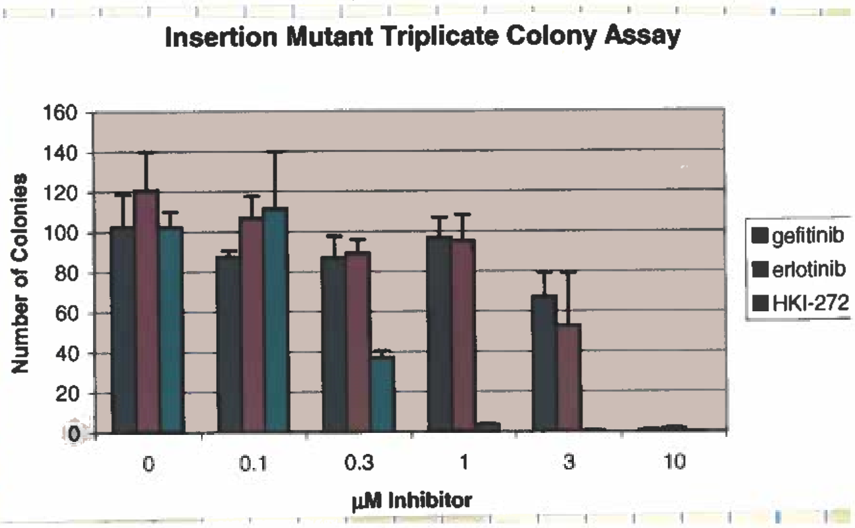

Supplement: S14 File — ins124 = EGFR insNPG (PDF) [file pmed.1004470.s014.pdf]
