# Supplementary material for: Correction: Oncogenic Transformation by Inhibitor-Sensitive and -Resistant EGFR Mutants
Source: PLoS Med. 2024 Sep 16;21(9):e1004470. doi: 10.1371/journal.pmed.1004470 (PMC11405057; doi:10.1371/journal.pmed.1004470)
Supplement: S15 File — (PDF) [file pmed.1004470.s015.pdf]

② TH's Tarceva CP for additional insertion & del mutants

olyclonal

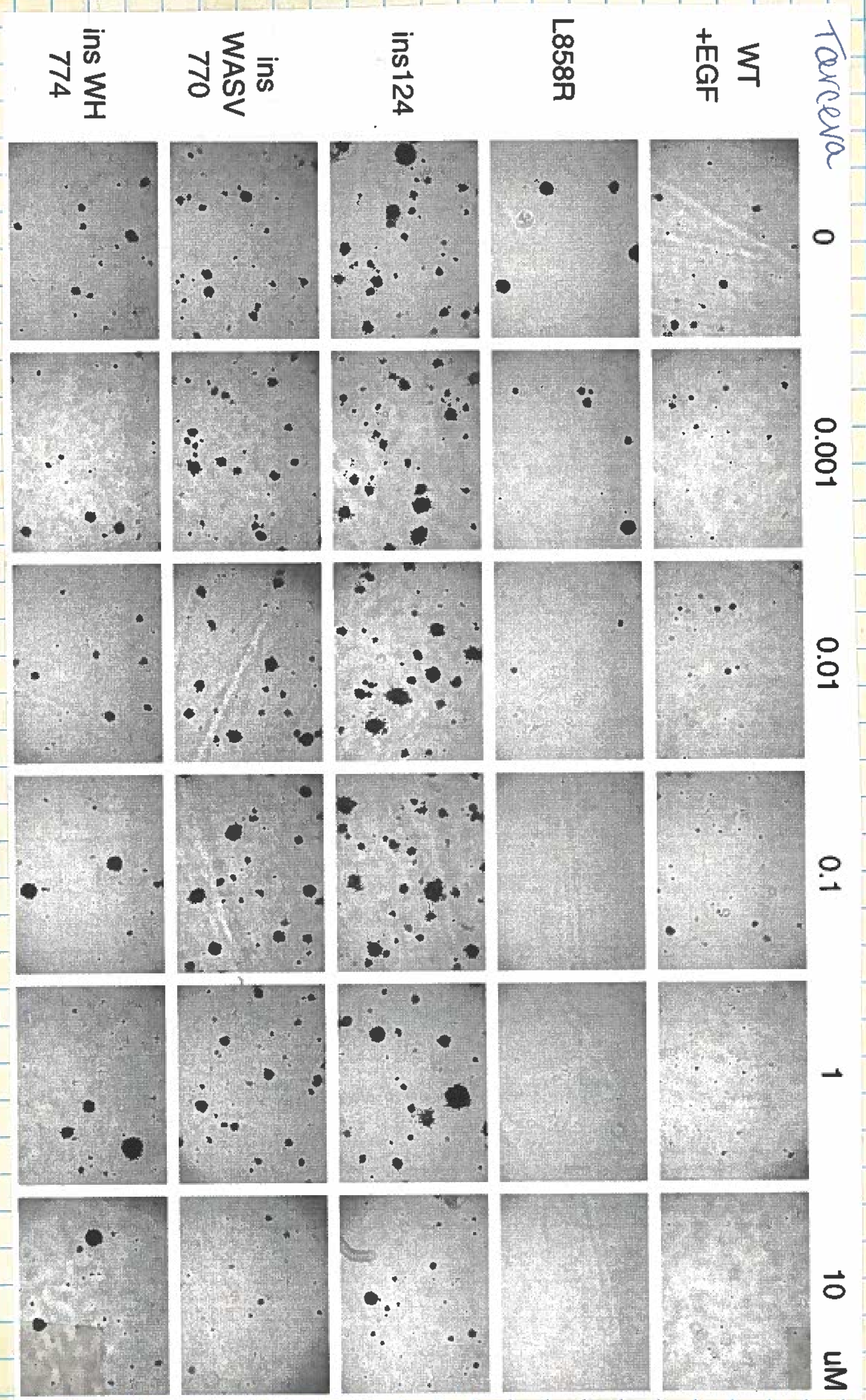

looks great! additional exon 20 ins mutants are resistant to Tarceva!
WASV = V769 D770insASV WH = H773 V774insH
